# The nuclear receptor RXRA controls cellular senescence by regulating calcium signaling

**DOI:** 10.1111/acel.12831

**Published:** 2018-09-14

**Authors:** Xingjie Ma, Marine Warnier, Clotilde Raynard, Mylène Ferrand, Olivier Kirsh, Pierre‐Antoine Defossez, Nadine Martin, David Bernard

**Affiliations:** ^1^ Centre de Recherche en Cancérologie de Lyon, Inserm U1052, CNRS UMR 5286, Centre Léon Bérard Université de Lyon Lyon France; ^2^ Epigenetics and Cell Fate, Sorbonne Paris Cité, CNRS UMR 7216 Université Paris Diderot Paris France

**Keywords:** calcium, ITPR2, nuclear receptor, p53, senescence

## Abstract

Calcium signaling is emerging as a key pathway controlling cellular senescence, a stable cell proliferation arrest playing a fundamental role in pathophysiological conditions, such as embryonic development, wound healing, cancer, and aging. However, how calcium signaling is regulated is still only partially understood. The inositol 1, 4, 5‐trisphosphate receptor type 2 (ITPR2), an endoplasmic reticulum calcium release channel, was recently shown to critically contribute to the implementation of senescence, but how ITPR2 expression is controlled is unclear. To gain insights into the regulation of ITPR2 expression, we performed an siRNA screen targeting 160 transcription factors and epigenetic regulators. Interestingly, we discovered that the retinoid X receptor alpha (RXRA), which belongs to the nuclear receptor family, represses ITPR2 expression and regulates calcium signaling though ITPR2 and the mitochondrial calcium uniporter (MCU). Knockdown of RXRA induces the production of reactive oxygen species (ROS) and DNA damage via the ITPR2‐MCU calcium signaling axis and consequently triggers cellular senescence by activating p53, whereas RXRA overexpression decreases DNA damage accumulation and then delays replicative senescence. Altogether, our work sheds light on a novel mechanism controlling calcium signaling and cellular senescence and provides new insights into the role of nuclear receptors.

## 1 INTRODUCTION

Cellular senescence is a permanent cell proliferation arrest induced in response to a wide variety of stresses, including telomere shortening, oxidative stress, DNA damage, and strong mitogenic signals. Senescent cells are mainly characterized by a stable cell cycle arrest and the appearance of features such as senescence‐associated‐β‐galactosidase activity (SA‐β‐Gal) and senescence‐associated secretory phenotype (SASP; Kuilman, Michaloglou, Mooi, & Peeper, 2010). Senescence plays a key role in many pathophysiological contexts, such as embryonic development, wound healing, cancer, and aging (He, & Sharpless, 2017). Understanding how senescence is controlled at the molecular and cellular level is therefore crucial. The senescence‐associated cell cycle arrest is mainly induced by p53 and its downstream target p21, by the cyclin‐dependent kinase inhibitor p16 and ultimately by RB which represses pro‐proliferative E2F target genes (Salama, Sadaie, Hoare, & Narita, 2014). Interestingly, calcium signaling is also emerging as a key player in the implementation of cellular senescence (Martin, & Bernard, 2017).

Intracellular calcium levels have been shown to increase in various cell types in response to senescence‐inducing stresses (Borodkina et al., 2016; Wiel et al., 2014; Yu, Li, et al., 2013; Yu, Zhang, Zhou, Yao, & Li, 2013) and activation of the calcium‐dependent transcription factor nuclear factor of activated T cells (NFAT) by the calcium/calmodulin/calcineurin pathway is known to regulate senescence (Manda et al., 2016; Wu et al., 2010). However, how calcium signaling is controlled is still only partially understood. We recently discovered that the inositol 1, 4, 5‐trisphosphate receptor type 2 (ITPR2), an endoplasmic reticulum (ER) calcium release channel, critically contributes to the implementation of cellular senescence (Wiel et al., 2014). Whereas ITPR2 activity has been shown to be modulated through protein interaction and post‐translational modification (Vervloessem, Yule, Bultynck, & Parys, 2015), how ITPR2 is regulated at the expression level is unclear. To address this question, we performed a genetic screen for regulators of ITPR2 and thereby identified the retinoid X receptor alpha (RXRA) as a transcriptional repressor of ITPR2.

RXRA is part of the nuclear receptor (NR) superfamily. NRs mainly function as transcriptional regulators, controlling organismal development, homeostasis, and metabolism (Sever, & Glass, 2013). More particularly, RXRA belongs to the type II receptors, which reside in the nucleus bound to DNA and repress the expression of their target genes in the absence of ligand through interaction with corepressor complexes (Evans, & Mangelsdorf, 2014; Sever, & Glass, 2013). RXRA is known to be implicated in various biological processes, including cell differentiation, cell death, and lipid metabolism (Evans, & Mangelsdorf, 2014; Szanto et al., 2004). However, no link between RXRA and calcium signaling has been reported so far. In this study, we show that RXRA modulates calcium signaling by directly regulating ITPR2 expression and thereby controls cellular senescence.

## 2 RESULTS

### 2.1 An siRNA screen identifies the nuclear receptor RXRA as a transcriptional repressor of *ITPR2*

**Figure 1:**
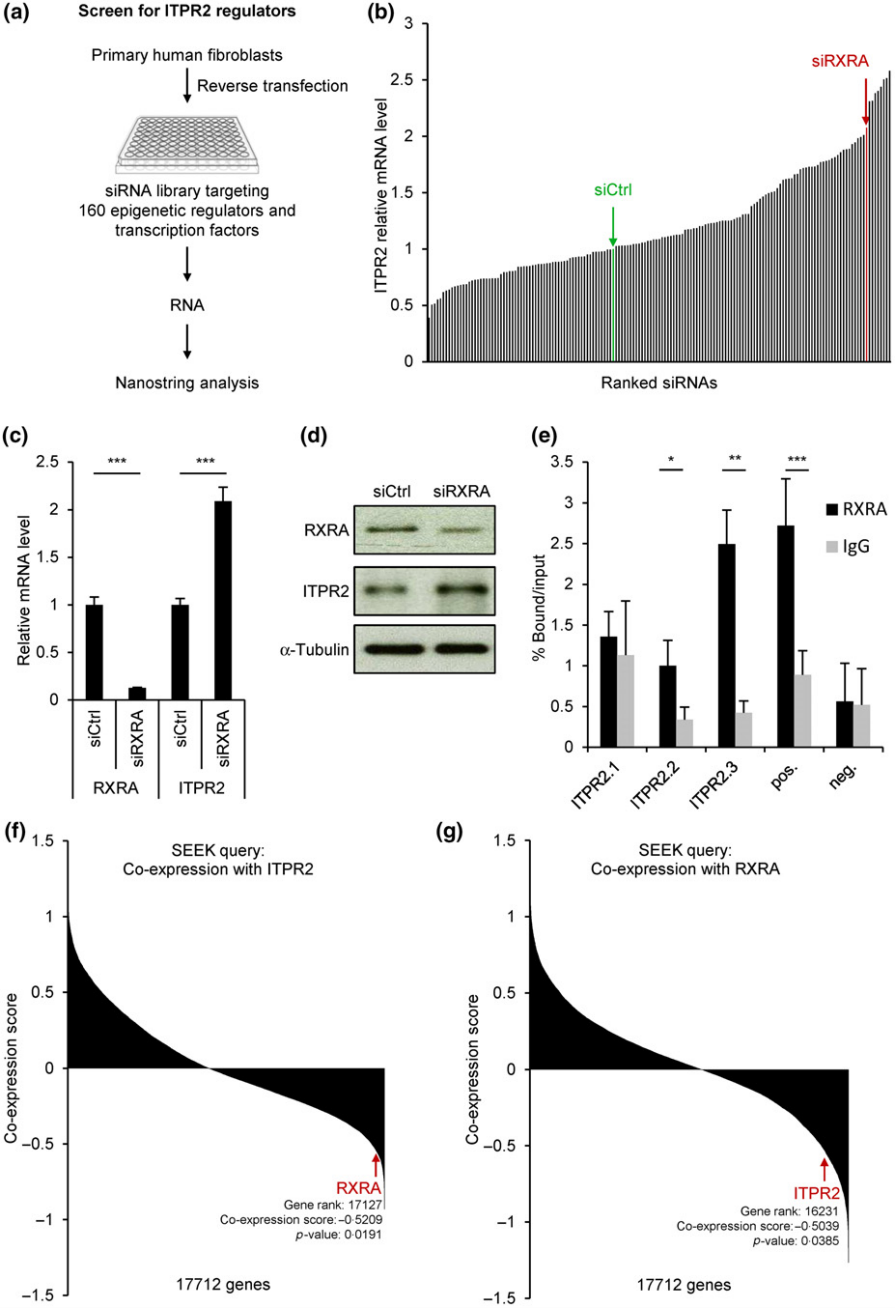
The nuclear receptor RXRA represses *ITPR2* expression. (a) Scheme depicting the screen for regulators of *ITPR2* expression. IMR90 primary human lung fibroblasts were transfected with an siRNA library containing a control nontargeting siRNA pool (siCtrl) and 160 siRNA pools each targeting an epigenetic regulator or a transcription factor with four different siRNAs. Four days after transfection, total RNA was prepared and ITPR2 mRNA level was quantified by Nanostring technology. (b) Results of the screen. siRNAs were ranked according to ITPR2 mRNA level compared to siCtrl. RXRA is one of the top repressors of ITPR2. (c) MRC5 primary human lung fibroblasts were transfected with a control nontargeting siRNA pool (siCtrl) or an siRNA pool targeting RXRA (siRXRA). Four days after transfection, RXRA and ITPR2 mRNA levels were quantified by RT–qPCR. (d) Six days after MRC5 transfection with siCtrl or siRXRA, RXRA and ITPR2 protein levels were analyzed by Western blot. α‐Tubulin was used as loading control. (e) Chromatin immunoprecipitation with RXRA antibody or control IgG was performed in MRC5 cells. The enrichment of bound chromatin fragments was quantified by qPCR either with primers amplifying regions of ITPR2 showing RXRA binding in ENCODE ChIP‐seq database (ITPR2), with primers amplifying a region described as a RXRA target (Varin et al., 2015) and used as positive control (pos.) or with primers amplifying a region showing no RXRA binding in ENCODE and used as negative control (neg.). See Supporting Information Figure S2 for further details on these regions. The experiments shown in panels c to e are representative of at least three biological replicates. Values are mean ± *SD*, and statistical analyses were performed with Student’s *t* test (*p < 0.05, **p < 0.01, ***p < 0.001). (f, g) The SEEK co‐expression database was interrogated for genes co‐expressed with ITPR2 (f) or RXRA (g) in lung noncancer samples.

To identify proteins controlling *ITPR2* expression, we screened a library of 160 pools of four siRNAs targeting epigenetic regulators and transcription factors. *ITPR2* expression was analyzed using the Nanostring technology (Figure 1a, Supporting Information Figure S1a, and Table S1). This approach allowed us to identify several candidates which knockdown modulates *ITPR2* expression (Figure 1b, Supporting Information Table S1). Among them, 10 genes were able to upregulate *ITPR2* expression by at least two fold when knocked down (Figure 1b, Supporting Information Table S1). We retested the effect of some of these potential *ITPR2* gene repressors (based on their novelty in term of senescence regulation and/or calcium regulation) in three different strains of primary human lung fibroblasts (IMR90, WI38 and MRC5) and observed the most significant and reproducible upregulation of *ITPR2* expression by knocking down the gene encoding the nuclear receptor RXRA (Supporting Information Figure S1b–d). We confirmed both at the mRNA and protein levels that RXRA knockdown induces *ITPR2* expression (Figure 1c,d). A similar observation was made using either a pool of four siRNAs targeting siRNAs or two individual siRNAs targeting RXRA (Supporting Information Figure S2a). As RXRA functions as a transcriptional repressor (Evans, & Mangelsdorf, 2014), we next asked whether RXRA could directly regulate *ITPR2*. We interrogated the Encyclopedia of DNA Elements (ENCODE) ChIP‐seq database and observed three peaks of RXRA on intron 2 of *ITPR2* in one dataset (Supporting Information Figure S2b‐c). In MRC5 fibroblasts, RXRA was found to preferentially bind the region 3 of *ITPR2* (Figure 1e and Supporting Information Figure S2b,c). Altogether, these data indicate that the nuclear receptor RXRA directly represses *ITPR2* expression.

To test the relevance of this finding, we searched the Search‐based exploration of expression compendium (SEEK) co‐expression database and found out that RXRA is among the genes which expression is the most significantly inversely correlated with the one of *ITPR2* in lung noncancer samples from 63 datasets (Figure 1f). Conversely, *ITPR2* was found among the genes which expression is the most significantly inversely correlated with the one of RXRA in these samples (Figure 1g). These data are consistent with a repression of *ITPR2* by RXRA.

### 2.2 RXRA regulates calcium release from the endoplasmic reticulum and accumulation in the mitochondria

**Figure 2:**
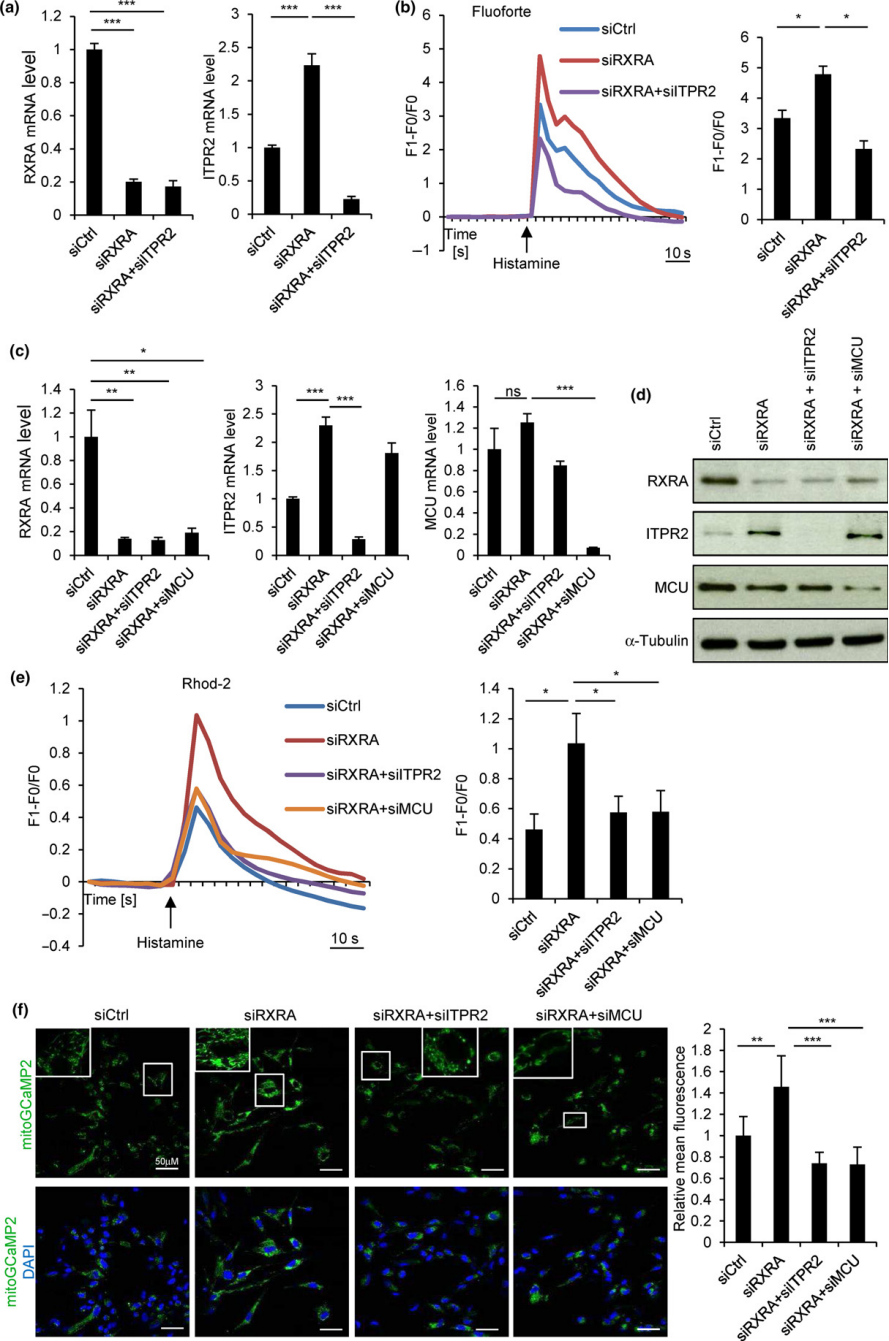
RXRA regulates calcium fluxes. (a) MRC5 were transfected with a control nontargeting siRNA pool (siCtrl) or with siRNA pools targeting RXRA or ITPR2 as indicated. Four days later, knockdown efficiency was checked by RT–qPCR. (b) Six days after transfection, live cells were charged with cytosolic calcium indicator Fluoforte, treated with 100 µM histamine, and fluorescence was analyzed by confocal microscopy. Fluorescence intensity over time (left) and at the maximum (right) is shown. (c) MRC5 were transfected with siCtrl pool or with siRNA pools targeting RXRA, ITPR2, or MCU as indicated. Four days later, knockdown efficiency was checked by RT–qPCR. (d) Six days after transfection, RXRA, ITPR2, and MCU protein levels were analyzed by Western blot. α‐Tubulin was used as loading control. (e) Six days after transfection, live cells were charged with mitochondrial calcium indicator Rhod‐2, treated with 100 µM histamine, and fluorescence was recorded by confocal microscopy. Fluorescence intensity over time (left) and at the maximum (right) is shown. (f) MRC5 infected with a retroviral vector encoding the mitochondrial calcium genetic reporter mitoGCaMP2 were transfected with siCtrl pool or with siRNA pools targeting RXRA, ITPR2, or MCU as indicated. Six days after transfection, fluorescence was recorded by confocal microscopy. Representative pictures (left) and fluorescence quantification (right) are shown. All the experiments shown are representative of at least three biological replicates. Statistical analysis was performed with Student’s *t* test (*p < 0.05, **p < 0.01, ***p < 0.001).

As ITPR2 is an endoplasmic reticulum (ER) calcium release channel, we next investigated whether RXRA, by repressing *ITPR2* expression, regulates calcium fluxes. Using the cytosolic calcium indicator Fluoforte and calcium imaging, we observed that RXRA knockdown by a siRNA pool or two individual siRNAs increased histamine‐induced calcium release from the ER (Figure 2a,b and Supporting Information Figure S3a) and that ITPR2 knockdown (Figure 2a) impaired this effect (Figure 2b).

As calcium released by the ER can be transferred to mitochondria (de Brito, & Scorrano, 2010; Hayashi, Rizzuto, Hajnoczky, & Su, 2009), the impact of RXRA on mitochondrial calcium accumulation was then investigated using the Rhod‐2 mitochondrial calcium indicator. RXRA knockdown by a siRNA pool or two individual siRNAs triggered an increase in mitochondrial calcium accumulation induced by histamine (Figure 2c–e and Supporting Information Figure S3b), and this was dependent on ITPR2 (Figure 2c–e). When assessing the involvement of the mitochondrial calcium uniporter (MCU), which mediates calcium entry in mitochondria (de Brito, & Scorrano, 2010), we observed that *MCU* expression was not significantly regulated by RXRA (Figure 2c,d) but that MCU was contributing to the increased mitochondrial calcium accumulation triggered by RXRA knockdown upon histamine stimulation (Figure 2c–e).

To measure basal mitochondrial calcium concentration, we introduced mitoGCaMP2, a genetically encoded mitochondria‐targeted calcium indicator (Chen et al., 2011), in MRC5 fibroblasts by retroviral infection. A higher concentration of calcium in the mitochondria was observed upon RXRA knockdown, and this increase was prevented when ITPR2 or MCU was concomitantly knocked down (Figure 2f). Altogether, these data indicate that RXRA regulates calcium release from the ER through ITPR2 and calcium accumulation in the mitochondria through MCU.

### 2.3 RXRA controls ROS production and DNA damage through calcium signaling

**Figure 3:**
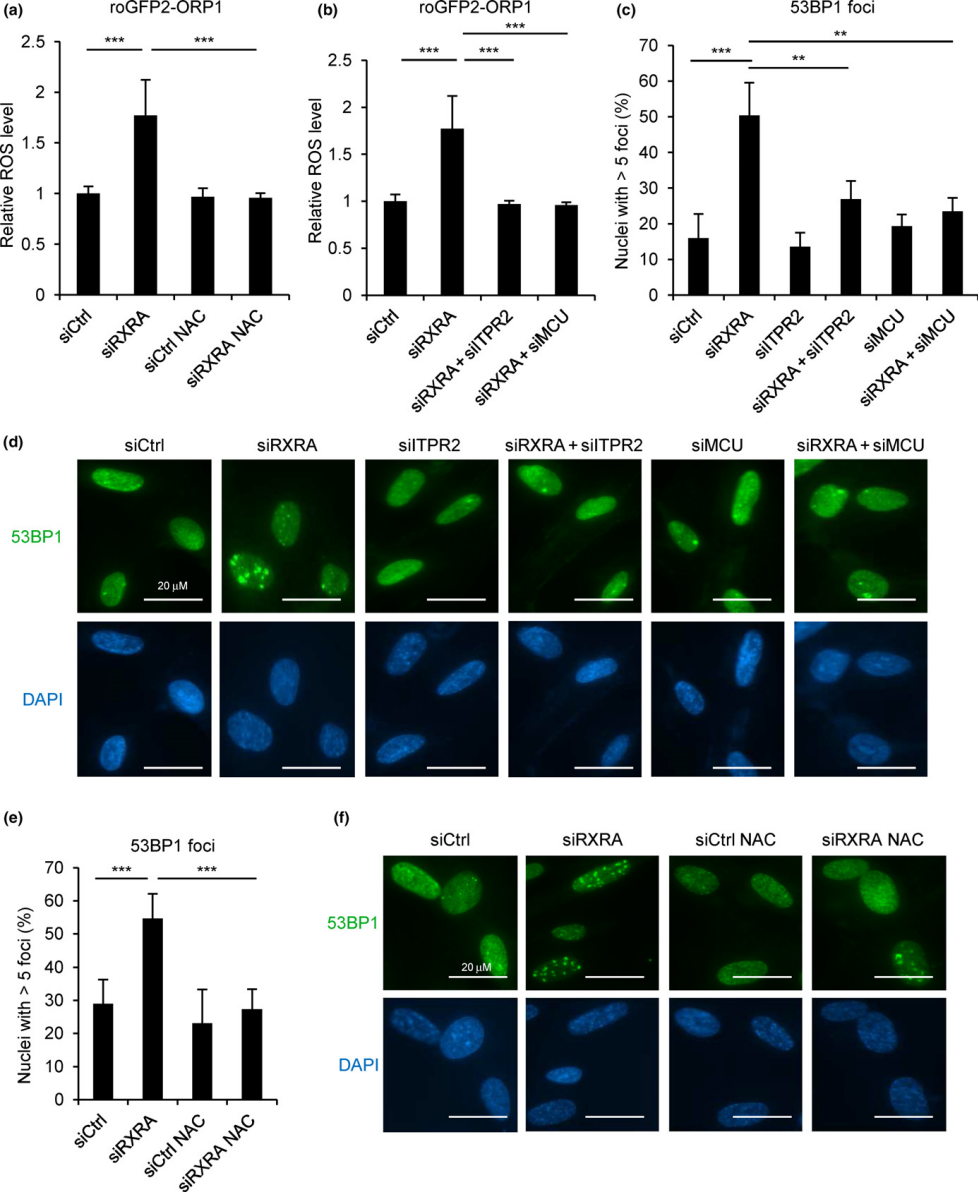
RXRA knockdown triggers ROS production and DNA damage. (a) MRC5 cells infected with a retroviral vector encoding the ROS reporter roGFP2‐ORP1 were transfected with a control nontargeting siRNA pool (siCtrl) or with a siRNA pool targeting RXRA and were then treated with the NAC antioxidant (1 µM) every 2 days where indicated. Six days after transfection, fluorescence was analyzed by confocal microscopy. (b) MRC5 cells stably expressing the ROS reporter roGFP2‐ORP1 were transfected with siCtrl pool or with siRNA pools targeting RXRA, ITPR2, or MCU as indicated. Fluorescence was analyzed by confocal microscopy 6 days after transfection. (c, d) MRC5 cells were transfected with siCtrl pool or with siRNA pools targeting RXRA, ITPR2, or MCU as indicated. Six days after transfection, immunofluorescence staining using anti‐53BP1 antibody was performed. Quantification of nuclei with more than five 53BP1 foci is shown (c) as well as representative pictures (d). (e, f) MRC5 cells were transfected with siCtrl or siRXRA pools and then treated with the NAC antioxidant (1 µM) every 2 days where indicated. Immunofluorescence staining of 53BP1 was performed 6 days after transfection. Quantification of nuclei with more than five 53BP1 foci is shown (e) as well as representative pictures (f). All the experiments shown are representative of at least three biological replicates. Statistical analysis was performed with Student’s *t* test (**p < 0.01, ***p < 0.001).

Mitochondrial calcium accumulation has been shown to trigger the generation of reactive oxygen species (ROS; Gorlach, Bertram, Hudecova, & Krizanova, 2015). Therefore, we hypothesized that RXRA knockdown, by inducing calcium accumulation in the mitochondria, could cause ROS production. To monitor ROS, we introduced the ROS indicator roGFP2‐ORP1 (Gutscher et al., 2009) by retroviral infection in MRC5 fibroblasts. RXRA knockdown in these cells by a siRNA pool or two individual siRNAs triggered the generation of ROS (Figure 3a and Supporting Information Figure S4a), which was prevented by treating cells with N‐acetyl‐l‐cysteine (NAC), an antioxidant (Figure 3a). This production of ROS was dependent on ITPR2 and MCU (Figure 3b).

As ROS are known to cause DNA damage (Cadet, & Wagner, 2013), we then performed immunofluorescence staining of 53BP1 foci, which mark sites of DNA damage (Schultz, Chehab, Malikzay, & Halazonetis, 2000). RXRA knockdown by a siRNA pool or two individual siRNAs induced an increase in the number of 53BP1 foci (Figure 3c,d and Supporting Information Figure S4b), which was prevented by knocking down ITPR2 or MCU together with RXRA (Figure 3c,d). By treating cells with NAC (Figure 3e,f) or Trolox (6‐hydroxy‐2,5,7,8‐tetramethylchroman‐2‐carboxylic acid), another antioxidant (Supporting Information Figure S5), we observed that DNA damage induced by RXRA knockdown was due to ROS.

**Figure 4:**
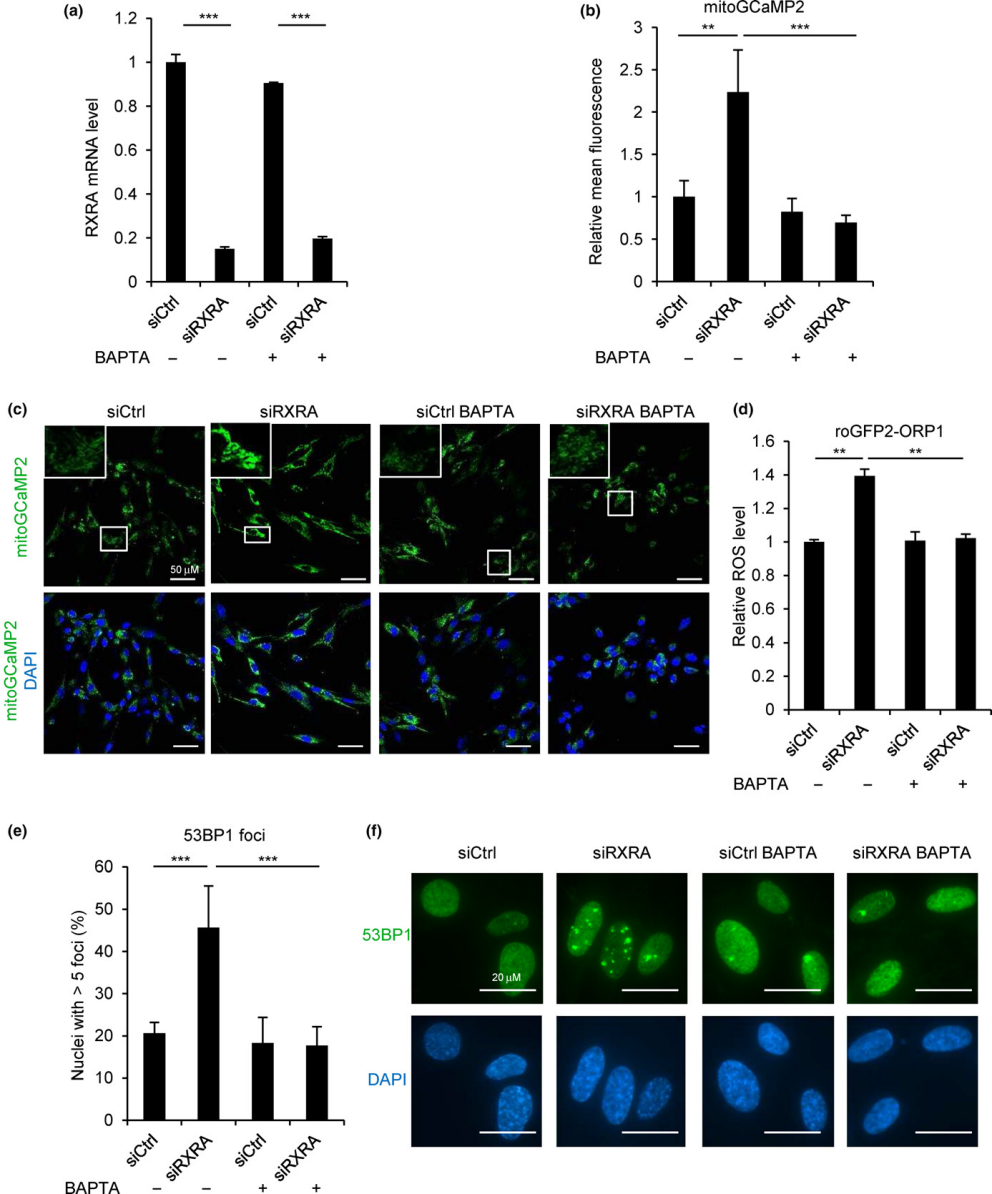
Calcium chelation prevents ROS production and DNA damage induced by RXRA knockdown. (a–c) MRC5 cells stably expressing the mitochondrial calcium indicator mitoGCaMP2 were transfected with a control nontargeting pool (siCtrl) or with a siRNA pool targeting RXRA and then treated with the intracellular calcium chelator BAPTA (1 µM) every 2 days where indicated. Four days after transfection, RXRA mRNA level was checked with RT–qPCR (a). Fluorescence was analyzed by confocal microscopy 6 days after transfection. Fluorescence quantification (b) and representative pictures (c) are shown. (d) MRC5 cells stably expressing the ROS reporter roGFP2‐ORP1 were transfected with siCtrl or siRXRA pools and were then treated with 1 µM BAPTA every 2 days where indicated. Six days after transfection fluorescence was analyzed by confocal microscopy. (e, f) MRC5 cells were transfected with siCtrl or siRXRA pools and then treated with BAPTA (1 µM) every 2 days where indicated. 53BP1 staining by immunofluorescence was performed 6 days after transfection. Quantification of nuclei with more than five 53BP1 foci is shown (e) as well as representative pictures (f). The experiments shown are all representative of at least three biological replicates. Statistical analysis was performed with Student’s *t* test (**p < 0.01, ***p < 0.001).

To assess whether calcium signaling is mediating these effects of RXRA, we used the intracellular calcium chelator BAPTA in RXRA knockdown experiments (Figure 4a). Calcium chelation not only prevented calcium accumulation in the mitochondria as expected (Figure 4b,c), but also prevented ROS production (Figure 4d) and DNA damage (Figure 4e,f) induced by RXRA knockdown. Altogether, these data indicate that RXRA, by regulating calcium signaling though ITPR2 and MCU, controls ROS production and consequently DNA damage.

### 2.4 RXRA knockdown triggers p53‐dependent cellular senescence through ITPR2 and MCU

**Figure 5:**
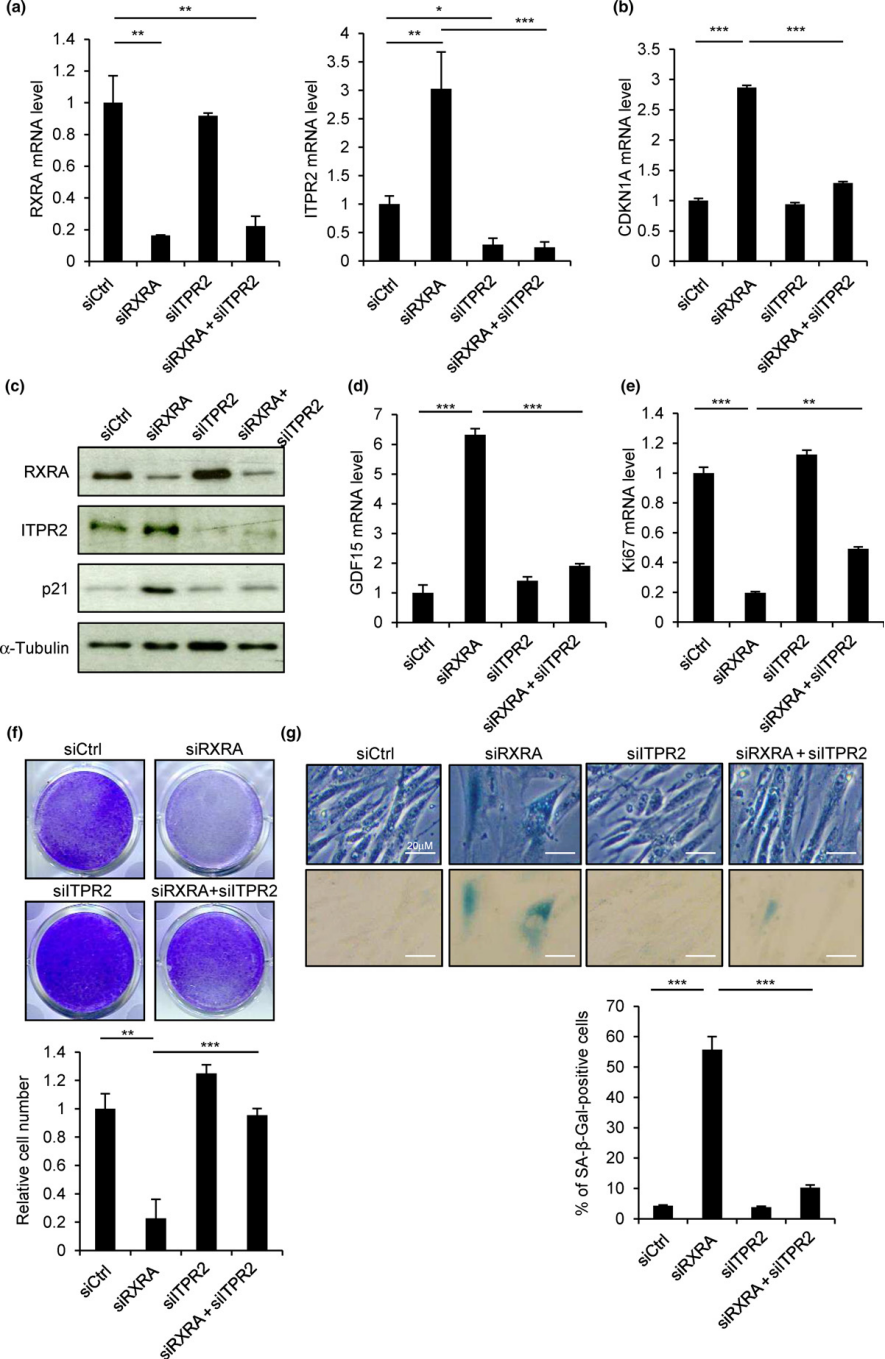
Cellular senescence induced by RXRA knockdown depends on ITPR2. MRC5 cells were transfected with a control nontargeting pool (siCtrl) or siRNA pools targeting RXRA or ITPR2 as indicated. (a–c) Four days after transfection, mRNA levels of RXRA and ITPR2 (a) and CDKN1A (b) were checked by RT–qPCR. (c) Six days after transfection, RXRA, ITPR2, and p21 protein levels were analyzed by Western blot. α‐Tubulin was used as loading control. (d, e) mRNA levels of GDF15 (d) and Ki‐67 (e) were also measured by RT–qPCR 4 days after transfection. (f) Six days after transfection, cells were stained with crystal violet (upper panel) and counted (lower panel). (g) SA‐β‐galactosidase assay was also performed 6 days after transfection. Representative pictures are shown (upper panel) as well as the percentage of SA‐β‐galactosidase‐positive cells counted in each condition (lower panel). All the experiments shown are representative of at least three biological replicates. Statistical analysis was performed with Student’s *t* test (*p < 0.05, **p < 0.01, ***p < 0.001).

As DNA damage activates p53 (Lakin, & Jackson, 1999), we then tested the effect of RXRA knockdown (Figure 5a) on the expression of the p53 target gene *CDKN1A*, which encodes the cyclin‐dependent kinase inhibitor p21. An increase in *CDKN1A* mRNA level (Figure 5b) and p21 protein level (Figure 5c) was observed. By contrast, RXRA knockdown did not alter the expression of *CDKN1B*, *CDKN2A*, and *CDKN2B* encoding other cyclin‐dependent kinase inhibitors (Supporting Information Figure S6a,b). RXRA knockdown also upregulated the expression of *GDF15*, a p53 target gene encoding a protein of the SASP (Figure 5d). A significant induction of the expression of several other genes encoding SASP components was observed (BMP2, COL3A1, IGFBP5, MMP3, PDGFA, VEGFA; Supporting Information Figure S6c). Interestingly, PDGFA and COL3A1 were recently described as part of the distinct SASP driven by NOTCH1 (Hoare et al., 2016). Furthermore, RXRA knockdown decreased cell proliferation as we observed a drop in the expression of the cell proliferation marker Ki‐67 (Figure 5e and Supporting Information Figure S7) and a decrease in the number of cells (Figure 5f). Importantly, RXRA knockdown also caused an increased SA‐β‐galactosidase activity (Figure 5g). Similar observations were made using either the siRXRA pool or individual siRNAs targeting RXRA, and both in MRC5 (Supporting Information Figure S8) and in IMR90 cells (Supporting Information Figure S9). Moreover, these effects of RXRA knockdown were rescued by overexpressing RXRA (Supporting Information Figure S10). Altogether, these data indicate that RXRA knockdown triggers cellular senescence.

To assess whether this senescent phenotype was dependent on ITPR2, we knocked down ITPR2 together with RXRA (Figure 5a). ITPR2 knockdown impaired the upregulation of *CDKN1A* (Figure 5b,c) and *GDF15* (Figure 5d). The proliferation arrest (Figure 5e,f and Supporting Information Figure S7) and the increase in SA‐β‐galactosidase activity (Figure 5g) triggered by RXRA knockdown were also impaired. Similar observations were made using either a siITPR2 pool or individual siRNAs targeting ITPR2 (Supporting Information Figure S11). Using the same approach, we further showed that cellular senescence induced by RXRA knockdown was also dependent on MCU (Supporting Information Figure S12) and p53 (Supporting Information Figure S13). Altogether, these data indicate that RXRA knockdown triggers p53 activation and p53‐dependent cellular senescence through ITPR2 and MCU.

### 2.5 RXRA constitutive expression delays replicative senescence

**Figure 6:**
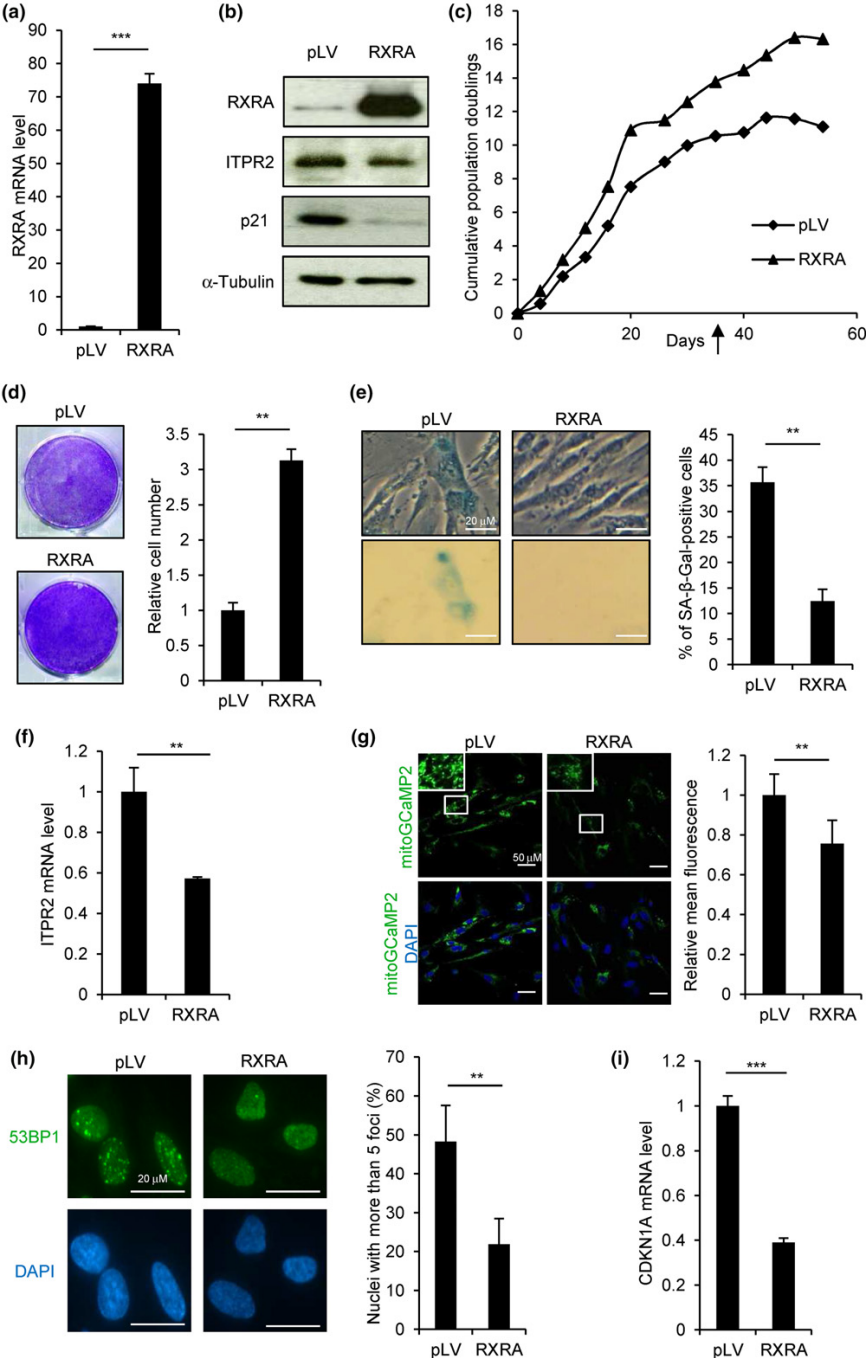
RXRA overexpression delays replicative senescence. MRC5 cells were infected with a lentiviral vector encoding RXRA (RXRA) or the corresponding empty vector as control (pLV). (a) RXRA mRNA level was checked by RT–qPCR. (b) RXRA, ITPR2, and p21 protein levels were analyzed by Western blot. α‐Tubulin was used as loading control. (c) Cells were counted at each passage, and cumulative population doublings were calculated. At the time point indicated by the arrow, the following assays were performed. (d) Cells were stained with crystal violet (left) and counted (right). (e) SA‐β‐galactosidase assay was performed. Representative pictures are shown (left) as well as the percentage of SA‐β‐galactosidase‐positive cells counted in each condition (right). (f) ITPR2 mRNA level was quantified by RT–qPCR. (g) MRC5 cells stably expressing the mitochondrial calcium indicator mitoGCaMP2 were infected with pLV or RXRA lentiviral vectors, and fluorescence was analyzed by confocal microscopy. Representative pictures (left) and fluorescence quantification (right) are shown. (h) 53BP1 was stained by immunofluorescence in MRC5 cells infected with pLV or RXRA vectors. Representative pictures are shown (left) as well as quantification of nuclei with more than five 53BP1 foci (right). (i) CDKN1A mRNA level was quantified by RT–qPCR. All the experiments shown are representative of at least two biological replicates. Statistical analysis was performed with Student’s *t* test (**p < 0.01, ***p < 0.001).

We then tested whether, conversely, RXRA overexpression impacts cellular senescence. RXRA was overexpressed in MRC5 primary human fibroblasts by lentiviral infection (Figure 6a,b), and the replicative potential of the cells was assessed. RXRA overexpression triggered an increase in the maximum number of population doublings (Figure 6c,d) and a decrease in SA‐β‐galactosidase activity (Figure 6e). In parallel, a reduction in *ITPR2* expression, in mitochondrial calcium concentration, in DNA damage, and in *CDKN1A* expression was observed (Figure 6b,f–i). Altogether, these data indicate that RXRA constitutive expression regulates the ITPR2–mitochondrial calcium–DNA damage–p21 axis and delays replicative senescence.

## 3 DISCUSSION

In this study, we identified the nuclear receptor RXRA as a novel regulator of calcium signaling and cellular senescence. Indeed, we observed that RXRA knockdown increases *ITPR2* expression and triggers calcium release from the ER and accumulation in the mitochondria. This calcium flux induces ROS production and DNA damage, which activates p53 and leads to cellular senescence through the p53‐p21 pathway. Conversely, RXRA overexpression decreases ITPR2 level, impairs the subsequent mitochondrial calcium–DNA damage–p21 signaling cascade, and delays replicative senescence.

Calcium signaling plays key roles in a number of biological processes including cellular senescence (Martin, & Bernard, 2017), but how calcium signaling is controlled is poorly understood. In particular, apart from the involvement of the calcineurin–NFAT pathway (Sankar, deTombe, & Mignery, 2014), only few things are known about the transcriptional regulation of *ITPR2* expression. Here, we identify RXRA as a transcriptional repressor of *ITPR2* and thus provide insights into ITPR2 regulation, which is important for cell fate decisions (Vervloessem et al., 2015). ENCODE ChIP‐seq data and our ChIP experiment point out RXRA as a direct regulator of *ITPR2*, although the identified RXRA binding site does not contain AGGTCA sequences which are the most frequent RXRA binding motifs (Evans, & Mangelsdorf, 2014; Sever, & Glass, 2013) and is located in the second intron of *ITPR2* rather than in its promoter. However, modulation of gene expression through the binding of transcriptional regulators in introns is more and more documented (Neph et al., 2012). Our work is the first to link RXRA with calcium signaling. We show that RXRA controls calcium release from the ER and accumulation in the mitochondria. As some calcium released from the ER is not transferred to the mitochondria but rather goes in the cytosol, RXRA might regulate calcium signaling in this compartment as well.

We also unveil that RXRA regulates cellular senescence as RXRA knockdown provokes premature senescence and RXRA overexpression delays replicative senescence. We investigated the effect of retinoic acid, an agonist of RXRA, and observed an upregulation of *ITPR2* expression (Supporting Information Figure S14). In line with these data, an increase in *ITPR2* expression was observed in several transcriptomic analyses upon treatment of cells with retinoic acid (Yu, Li, et al., 2013; Yu, Zhang, et al., 2013; Zheng et al., 2005). However, in our experimental conditions, we did not observe any effect of retinoic acid on cell proliferation (Supporting Information Figure S14), suggesting that removing the repressive RXRA transcriptional activity (knockdown) and activating RXRA transcriptional activity (retinoic acid treatment) are not functionally equivalent even if both result in *ITPR2* gene expression. Beyond identifying RXRA impact on senescence, we deciphered the mechanisms underlying it. We show that RXRA knockdown triggers senescence by activating the ITPR2/MCU/ROS/DNA damage/p53/p21 cascade, whereas RXRA constitutive expression displays opposite effects. This signaling pathway supports the published reports showing that calcium regulates ROS generation (Gorlach et al., 2015). Interestingly, RXRA knockdown was found to decrease cyclin D1 and PCNA expression (Huang et al., 2015), which could also contribute to the induction of cellular senescence.

A few studies have recently revealed that cellular senescence can be regulated by nuclear receptors (Graziano, Johnston, Deng, Zhang, & Gonzalo, 2016; Jin et al., 2016; O'Loghlen et al., 2015; Wu et al., 2015; Zambrano et al., 2014; Zhu et al., 2014). Nuclear receptors form a large family, and only a few of them have been implicated so far in cellular senescence. RXRA is an additional example showing the key role of nuclear receptors in this process. Of note, knockdown of the vitamin D receptor (VDR) has been recently shown to cause DNA damage and cellular senescence. DNA damage accumulation has been attributed to the capacity of VDR to control the levels of some DNA repair factors such as BRCA1 (Graziano et al., 2016). We unveil here an alternative pathway (calcium signaling and ROS) by which knockdown of nuclear receptors may promote DNA damage and cellular senescence.

In conclusion, our work sheds light on the link between nuclear receptors, calcium signaling, and cellular senescence. A challenge ahead will be to investigate the relevance of these observations in different contexts of cellular senescence, age‐related diseases, and aging.

## 4 MATERIALS AND METHODS

### 4.1 Cell culture and reagents

IMR90, WI38, and MRC5 human fetal lung fibroblasts (ATCC), GP‐293 retroviral, or 293 T lentiviral packaging cells (Clontech) were cultured in Dulbecco's modified Eagle's medium (DMEM, Life Technologies) containing GlutaMax and supplemented with 10% fetal bovine serum (FBS, Sigma‐Aldrich) and 1% penicillin/streptomycin (Life Technologies). Cells were maintained at 37°C under a 5% CO_2_ atmosphere. Cells were tested for mycoplasma and when needed treated with Plasmocin (Invivogen) until they became mycoplasma‐free before performing experiments. Experiments were performed in IMR90 at p14–p16, in WI38 at p21–24, and in MRC5 at p21–p25. NAC, Trolox, and 9‐cis‐retinoic acid were purchased from Sigma‐Aldrich and used at 1 µM, 50 µM, and 500 nM, respectively. BAPTA was purchased from Santa Cruz Biotechnology and used at 1 µM.

### 4.2 Vectors, transfection, and infection

pLNCX2‐mito‐GCaMP2 (Wiel et al., 2014) and pLPCx‐roGFP2‐ORP1 (Le et al., 2015) retroviral vectors were previously described. The lentiviral vector pLV‐RXRA and the corresponding empty vector pLV were purchased from Vectorbuilder. GeneJuice transfection reagent was used according to the recommendations of the manufacturer (Merck Millipore) to transfect GP‐293 cells with VSVg and the above‐mentioned retroviral vectors and 293 T cells with gag‐pol, env, and the above‐mentioned lentiviral vectors. Two days after transfection, the viral supernatant was mixed with fresh medium and 8 μg/mL hexadimethrine bromide (Sigma‐Aldrich) and used to infect MRC5 cells. One day later, infected cells were selected with 100 µg/ml neomycin (Life Technologies) or 500 ng/ml puromycin (Invivogen).

### 4.3 siRNA transfection

For the siRNA screen and other knockdown experiments, an ON‐TARGETplus nontargeting control pool (siCtrl), ON‐TARGETplus siRNA SMARTpools targeting the genes of interest with four different siRNAs and ON‐TARGETplus individual siRNAs were used (Dharmacon, GE Healthcare). Sequences of siRNAs comprised in the pools and of individual siRNAs are listed in Supporting Information Tables S2 and S3, respectively. Cells were reverse transfected with siRNAs using Dharmafect 1 transfection reagent according to manufacturer’s instructions (Dharmacon, GE Healthcare). siRNAs were used at 30 nM in the screen and at 15 nM in the following experiments. When transfecting two different siRNAs (pools or individuals) in the same well, each was transfected at 15 nM and siCtrl was used to keep the final concentration of siRNAs at 30 nM. Similar knockdown efficiencies were observed with these concentrations of siRNAs. Except if stated otherwise, reverse transfection was performed in 6‐well plates.

### 4.4 Screening and Nanostring analysis

For the screen, a library of 160 siRNA pools each targeting an epigenetic regulator or a transcription factor was used. The full list of the 160 genes targeted by the library is displayed in Supporting Information Table S1. The library was divided into four groups, and for each of them, siRNA pools and a control nontargeting siRNA pool (30 nM final concentration) were reverse transfected. Seventy thousand IMR90 cells were seeded per well in 6‐well plates for reverse transfection. Total RNAs were extracted 4 days after siRNA transfection using Upzol (Dutscher) and phenol–chloroform and were sent to the Genomics Platform of Institut Curie (Paris, France). RNAs were analyzed with the BioAnalyzer using Nano LabChip to assess their integrity (Bioanalyzer 2100 RNA 6000 Nano Kit from Agilent Technologies) and with Nanodrop (Thermo) to assess their purity and concentration. RNA abundance was then measured with Nanostring technology (Nanostring Flex nCounter analysis system). All RNA processed to analysis display a RIN > 7.6 and a ratio 260 nm/280 mm >1.8. Raw counts were first normalized for platform source of variations (Nanostring controls) and with three housekeeping genes (PGK1, TBP, and TUBB2A) as recommended by Nanostring technology, Inc. Nanostring probes are displayed in Supporting Information Table S4. Relative expression of *ITPR2* was calculated for each siRNA pool according to its level in cells transfected with the control nontargeting siRNA pool.

### 4.5 Reverse transcription and real‐time quantitative PCR

The Maxima first‐strand cDNA synthesis kit (Life Technologies) was used to synthesize cDNA from total RNA, according to manufacturer’s instructions. cDNAs obtained by this reverse transcription (RT) were used as templates for quantitative PCR (qPCR). The qPCR mixture also contained TaqMan mix (Roche), 100 µM of a Universal Probe Library probe (Roche), and 200 nM of primers (Sigma‐Aldrich). Primer sequences are listed in Supporting Information Table S5. qPCRs were carried out on a FX96 Thermocycler (Bio‐Rad). qPCRs were as follows: 95°C 10 min, followed with 40 cycles of 95°C 10 s, 59°C 30 s. The reactions were performed at least in duplicate. The relative amount of mRNA was calculated using the comparative Ct (ΔΔCt) method, and data were normalized using two housekeeping genes (PGK1 and HPRT1).

### 4.6 Western blot

Cells were directly lysed in Laemmli buffer. Cell lysates were resolved by SDS‐PAGE electrophoresis and transferred to nitrocellulose membranes (Bio‐Rad). Membranes were then blocked with TBST‐Milk 5% for 1 hr and incubated with primary antibodies overnight at 4°C. Primary antibodies and dilutions used are listed in Supporting Information Table S6. Horseradish peroxidase‐conjugated donkey anti‐rabbit and sheep anti‐mouse antibodies (Interchim) were used as secondary antibodies and incubated for 1 hr at room temperature. Detection was performed using ECL kit (Amersham).

### 4.7 Chromatin immunoprecipitation

Chromatin immunoprecipitation (ChIP) was performed with the Millipore ChIP Assay Kit (17‐295). MRC5 cells were crosslinked with 1% formaldehyde for 10 min at 37°C. Chromatin was prepared according to the Millipore protocol and sonicated to an average size of 300–500 bp using a Diagenode Bioruptor. Chromatin fragments were immunoprecipitated at 4°C overnight with RXRA antibody or normal rabbit IgG used as negative control (see references of antibodies in Supporting Information Table S6), and immune complexes were collected on Protein A agarose beads (ChIP assay kit, Millipore). Quantitative PCR was carried out using SYBR Green Supermix (Bio‐Rad). Primers amplifying ITPR2 regions showing RXRA binding in the ENCODE ChIP‐seq database were used, as well as primers for positive and negative controls. ENCODE data and location of primers that we used are presented in Supporting Information Figure S2. Primer sequences are indicated in Supporting Information Table S7. The amount of immunoprecipitated target DNA is represented as a percentage of input, which was calculated using a curve derived from serial dilutions of input chromatin.

### 4.8 SA‐β‐Galactosidase assay and crystal violet staining

For SA‐β‐galactosidase assay, cells were washed twice with PBS and fixed for 5 min in 2% formaldehyde/0.2% glutaraldehyde. Cells were then rinsed twice in PBS and incubated at 37°C overnight in SA‐β‐Gal solution as previously described (Dimri et al., 1995). For crystal violet staining, cells were washed with PBS, fixed for 15 min in 3.7% formaldehyde, and then stained with 0.5% crystal violet solution.

### 4.9 Calcium imaging

For Fluoforte and Rhod‐2 calcium assays, 6 days after siRNA transfection in glass‐bottom dishes (Thermo Scientific), live cells were treated either with 5 µM Fluoforte (ENZ‐52014, Enzo Life Sciences) or with 10 µM Rhod‐2 (R‐1245MP, Thermo Scientific) for 1 hr at 37 °C in Hank’s balanced salt solution (HBSS) with calcium, magnesium, and no phenol red (14025050, Thermo Scientific). Cells were then washed and incubated in HBSS with no calcium, no magnesium, and no phenol red (14175053, Thermo Scientific) for calcium imaging. Fluorescence was recorded every 1.5 s using Zeiss LSM 780 confocal microscope. Histamine (H7125‐1G, Sigma‐Aldrich) was injected after 1 min of measurement at a final concentration of 100 nM. Average changes in fluorescence intensities in multiple regions of interest (ROI) were calculated. Results are shown as (F1‐F0)/F0, where F0 is the mean of the intensities from 10 to 50 s. Alternatively, cells stably expressing mitoGCaMP2, a genetically encoded mitochondrially targeted calcium indicator, were transfected with siRNA in glass‐bottom dishes. Six days later, fluorescence (Ex 488 nm/Em 500–570 nm) was monitored in live cells using Zeiss LSM 780 confocal microscope. Fluorescence intensity was quantified using the ImageJ software.

### 4.10 ROS detection

Six days after siRNA transfection in glass‐bottom dishes (Thermo Scientific), cells stably expressing roGFP2‐ORP1 were washed and incubated in HBSS with calcium, magnesium, and no phenol red (14025050, Thermo Scientific) for ROS detection. Fluorescence (Ex 405 and 488 nm/Em 500–554 nm) was monitored in live cells using Zeiss LSM 780 confocal microscope. The ImageJ software was used to quantify fluorescence intensity, and the ratio between 405 nm (oxidized state) and 488 nm (reduced state) was plotted, as previously described (Gutscher et al., 2009).

### 4.11 Immunofluorescence staining

Six days after siRNA transfection in eight‐chamber tissue culture glass slides (Falcon, Corning), cells were fixed in ice‐cold methanol for 10 min at −20°C and blocked in PBS‐Tween 0.05% containing 20% FBS (PBST‐FBS). Incubation with primary antibodies in PBST‐FBS was performed overnight at 4°C. Primary antibodies and dilutions used are listed in Supporting Information Table S6. After three washes with PBS, the slides were incubated with Alexa Fluor 488 dye‐conjugated goat anti‐rabbit antibody or chicken anti‐mouse antibody diluted in PBST‐FBS for 1 hr at room temperature. The slides were then mounted with DAPI Fluoromount G (SouthernBiotech) after three PBS washes. Images were acquired with a Nikon fluorescence microscope, and data were collected and analyzed with NIS software (Nikon). For 53BP1 foci, their number per nucleus was determined using the Focinator tool (Oeck, Malewicz, Hurst, Rudner, & Jendrossek, 2015). More than 200 nuclei were analyzed for each condition.

### 4.12 Statistical analysis

Graphs are presented with *SD* as errors bars, and Student’s *t* test was used to determine the *p*‐value. *p < 0.05; **p < 0.01; ***p < 0.001.

## CONFLICT OF INTEREST

None declared.

## AUTHOR CONTRIBUTIONS

D.B. and N.M. conceived and supervised the project. D.B., N.M., and X.M. designed the experiments. X.M., M.F., M.W., C.R., and N.M. performed experiments. X.M., M.W., O.K., P.A.D., N.M., and D.B. performed data analysis. D.B., N.M., and X.M. wrote the manuscript.
