# Supplementary material for: The nuclear receptor RXRA controls cellular senescence by regulating calcium signaling
Source: Aging Cell. 2018 Sep 14;17(6):e12831. doi: 10.1111/acel.12831 (PMC6260923; doi:10.1111/acel.12831)
Supplement: Supplementary file 1 [file ACEL-17-e12831-s001.pdf]

Supplementary Figure 1, Ma et al

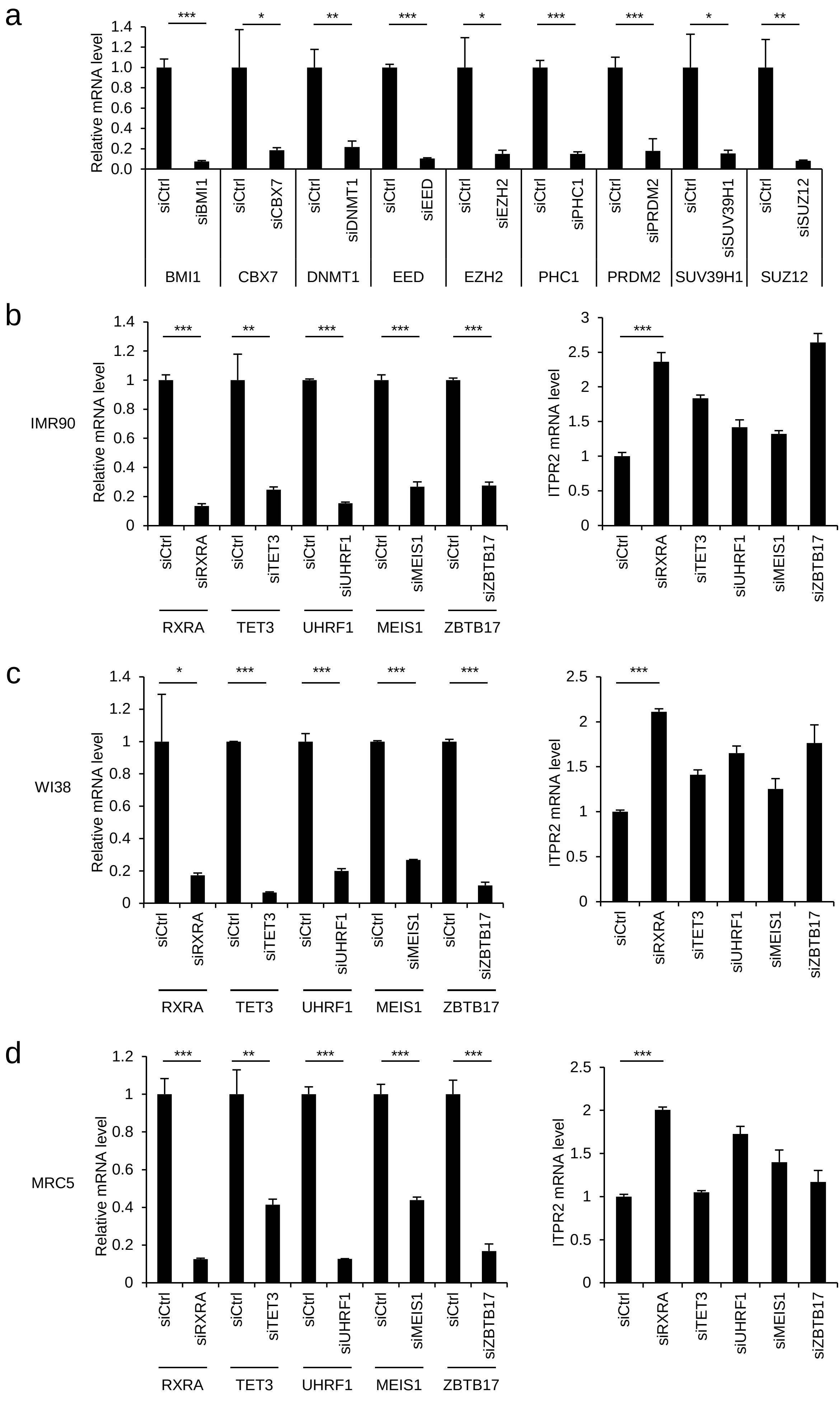

Supplementary Figure 1 An siRNA screen identifies RXRA as a regulator of ITPR2 expression. (a) 4 days after transfection of an siRNA library targeting 160 epigenetic regulators and transcription factors in IMR90 primary lung fibroblasts, RNA was extracted, and knockdown efficiency was checked by RT-qPCR for the indicated genes. mRNA levels in cells transfected with a control non-targeting siRNA pool (siCtrl) were used as reference. (b-d) siRNA pools targeting five of the genes which knockdown induces a significant upregulation of ITPR2 expression (Supplementary Table 1) were transfected in IMR90 (a), WI38 (b) and MRC5 (c), three different strains of primary human lung fibroblasts. Knockdown efficiency (left) and ITPR2 mRNA level (right) were analyzed by RT-qPCR. The experiments shown in panels b to d are representative of at least two biological replicates. Statistical analysis was performed with Student's t-test (* for $P < 0.05$, ** for $P < 0.01$, *** for $P < 0.001$).

Supplementary Figure 2, Ma et al

a

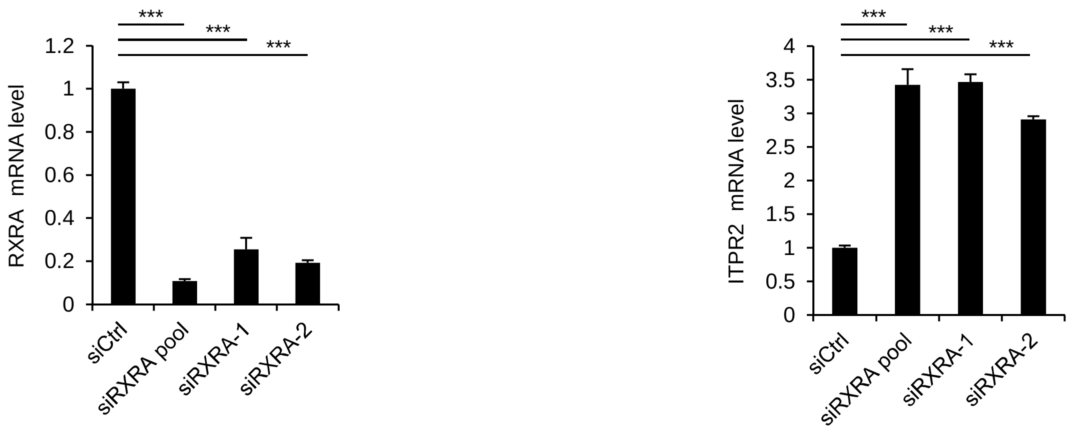

b

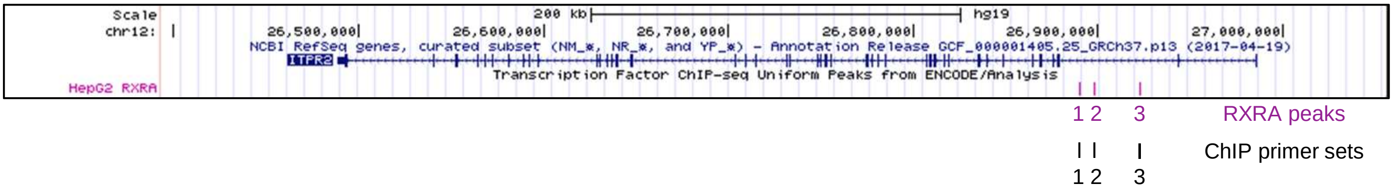

c

RXRA peaks on ITPR2 in ENCODE dataset	Location	Position
1	Intron 2 of *ITPR2*	chr12:26889789-26889960
2	Intron 2 of *ITPR2*	chr12:26897975-26898126
3	Intron 2 of *ITPR2*	chr12:26922225-26922374
**Primers used in ChIP experiments**	**Location**	**Position**
ITPR2.1	Intron 2 of *ITPR2*	chr12:26889738-26889967
ITPR2.2	Intron 2 of *ITPR2*	chr12:26897912-26898139
ITPR2.3	Intron 2 of *ITPR2*	chr12:26922141-26922370
Positive control	10 kb upstream of *ELOVL5* TSS	chr6:53224386-53224511
Negative control	Exon 1-Intron 1 of *CDKN1A*	chr6:36644243-36644536

Supplementary Figure 2 RXRA is a transcriptional repressor of ITPR2. (a) MRC5 primary human lung fibroblasts were transfected with a control non-targeting siRNA pool (siCtrl), a siRNA pool targeting RXRA (siRXRA pool) or individual siRNAs targeting RXRA (RXRA-1 and RXRA-2). 4 days after transfection, RXRA and ITPR2 mRNA levels were quantified by RT-qPCR. The experiments shown are representative of at least two biological replicates. Statistical analysis was performed with Student's t-test (*** for P <0.001). (b) Data from the ENCODE ChIP-seq database showing RXRA binding on ITPR2. ChIP-seq was performed by Myers-Hudson Alpha lab in HepG2 cells using sc-553 RXRA antibody from Santa Cruz Biotechnology (UCSC Accession: wgEncodeEH001506). RXRA peaks on ITPR2 and corresponding ChiP primers that we used for ChIP displayed in Figure 1e are indicated. (c) Location and position of RXRA peaks found on ITPR2 in the above-mentioned ENCODE dataset and of primers used in our ChIP experiments in Figure 1e (human genome GRCh37/hg19). A region upstream of ELOVL5 transcription start site previously described as a target of RXRA (Varin et al., 2015) was used as positive control.

Supplementary Figure 3, Ma et al

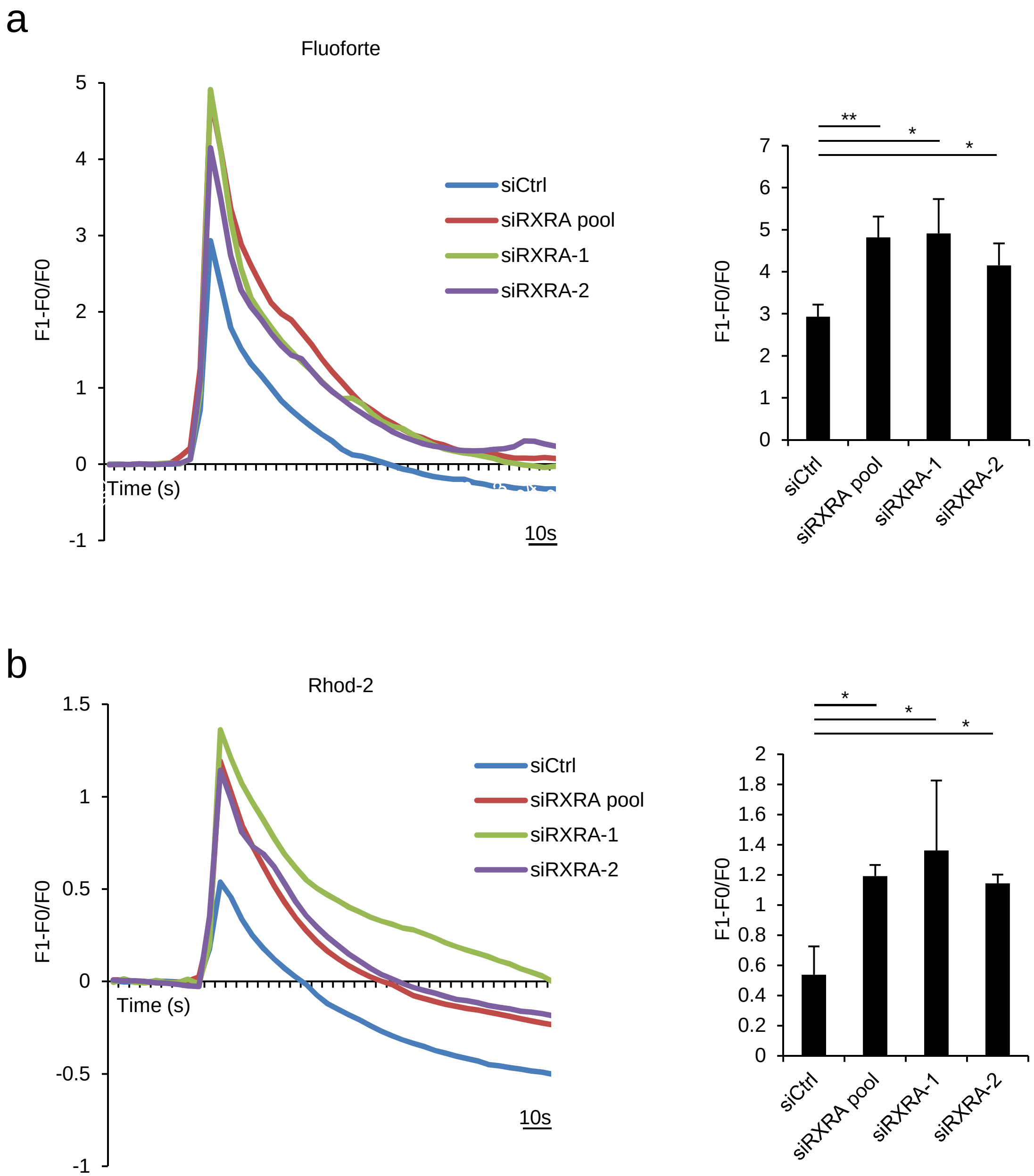

Supplementary Figure 3 RXRA regulates calcium fluxes. MRC5 were transfected with a control non-targeting siRNA pool (siCtrl), a siRNA pool targeting RXRA (siRXRA pool) or individual siRNAs targeting RXRA (RXRA-1 and RXRA-2). (a) 6 days after transfection, live cells were charged with cytosolic calcium indicator Fluoforte, treated with 100 µM histamine and fluorescence was analyzed by confocal microscopy. Fluorescence intensity over time (left) and at the maximum (right) is shown. (b) 6 days after transfection, live cells were charged with mitochondrial calcium indicator Rhod-2, treated with 100 µM histamine and fluorescence was recorded by confocal microscopy. Fluorescence intensity over time (left) and at the maximum (right) is shown. The experiments shown are representative of at least two biological replicates. Statistical analysis was performed with Student's t-test (* for P <0.05, ** for P <0.01).

Supplementary Figure 4, Ma et al

a

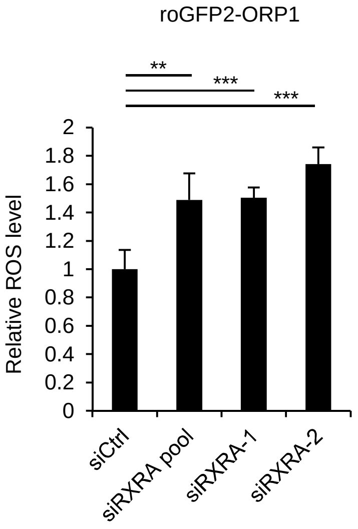

b

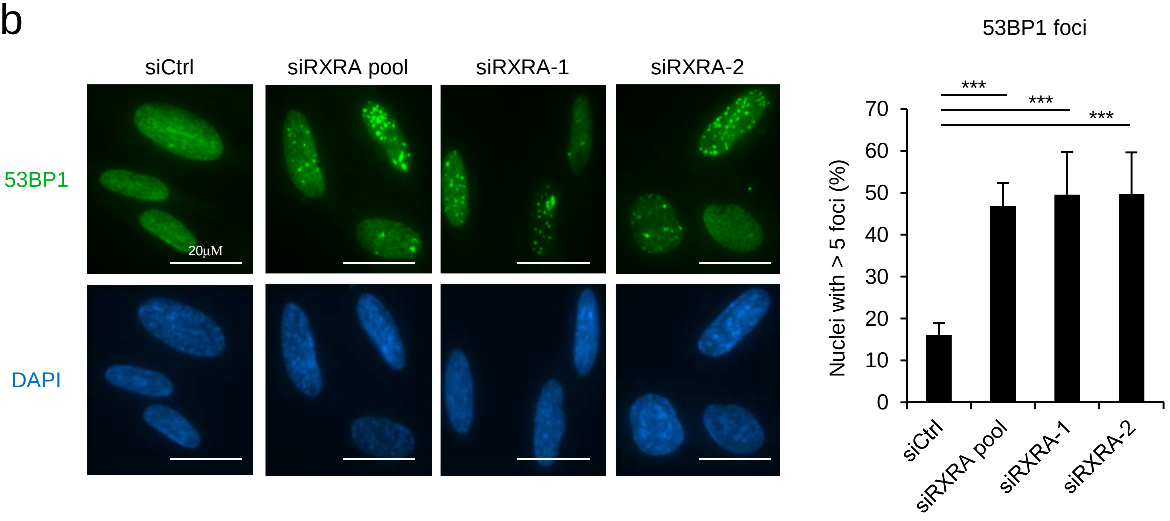

Supplementary Figure 4 RXRA knockdown triggers ROS production and DNA damage. (a) MRC5 cells infected with a retroviral vector encoding the ROS reporter roGFP2-Orp1 were transfected with a control non-targeting siRNA pool (siCtrl), a siRNA pool targeting RXRA (siRXRA pool) or individual siRNAs targeting RXRA (RXRA-1 and RXRA-2). 6 days after transfection, fluorescence was analyzed by confocal microscopy. (b) MRC5 cells were transfected with siCtrl pool, siRXRA pool or individual siRNAs RXRA-1 and RXRA-2. 6 days after transfection, immunofluorescence staining using anti-53BP1 antibody was performed. Representative pictures (left) are shown as well as quantification of nuclei with more than five 53BP1 foci (right). The experiments shown are representative of at least two biological replicates. Statistical analysis was performed with Student's t-test (** for P <0.01, *** for P <0.001).

Supplementary Figure 5, Ma et al

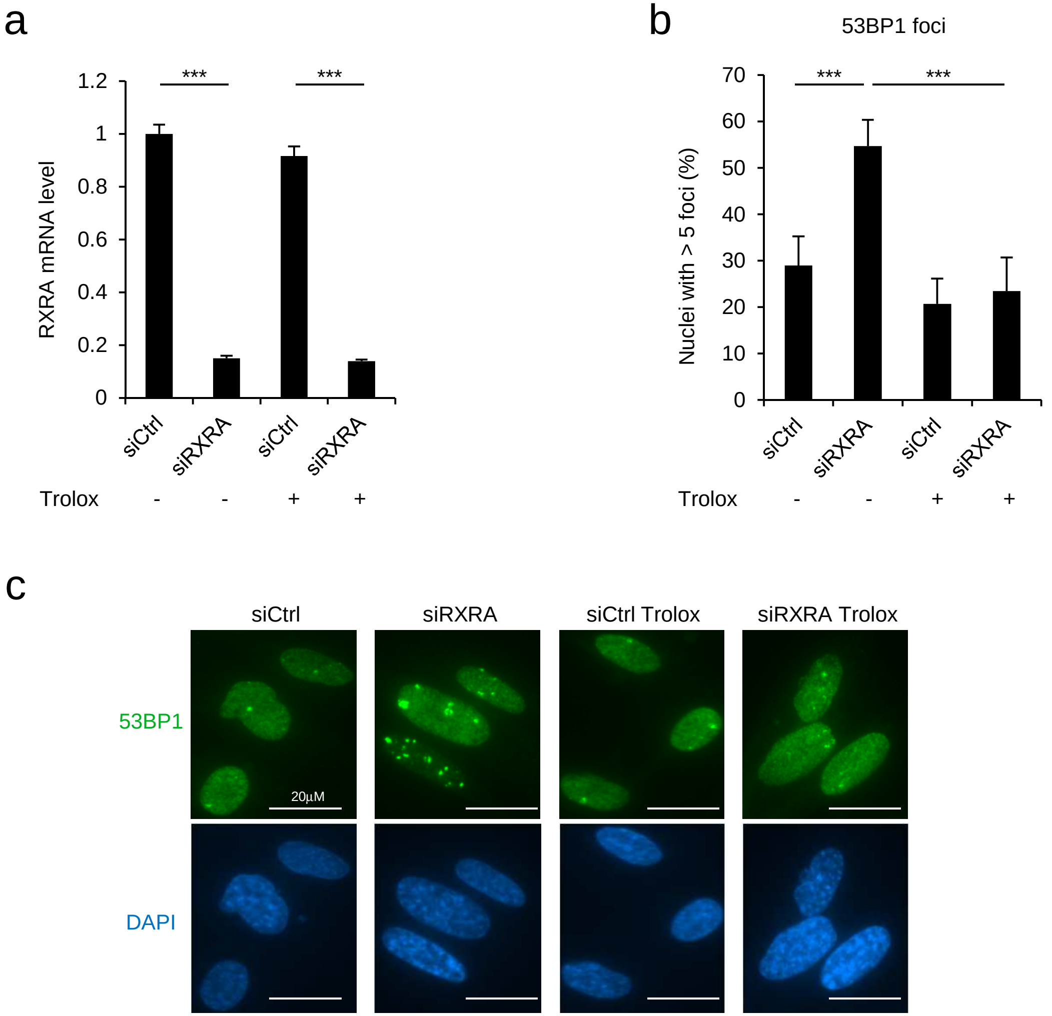

Supplementary Figure 5 Treatment with antioxidant prevents DNA damage induced by RXRA knockdown. MRC5 cells were transfected with a control non-targeting siRNA pool (siCtrl) or a siRNA pool targeting RXRA (siRXRA) and then treated with 50µM Trolox antioxidant every two days where indicated. (a) 4 days later, RXRA mRNA level was checked by RT-qPCR. (b-c) 6 days after transfection, immunofluorescence staining of 53BP1 was performed. Quantification of nuclei with more than five 53BP1 foci is shown (b) as well as representative pictures (c). The experiments shown are representative of at least three biological replicates. Statistical analysis was performed with Student's t-test (*** for P <0.001).

Supplementary Figure 6, Ma et al

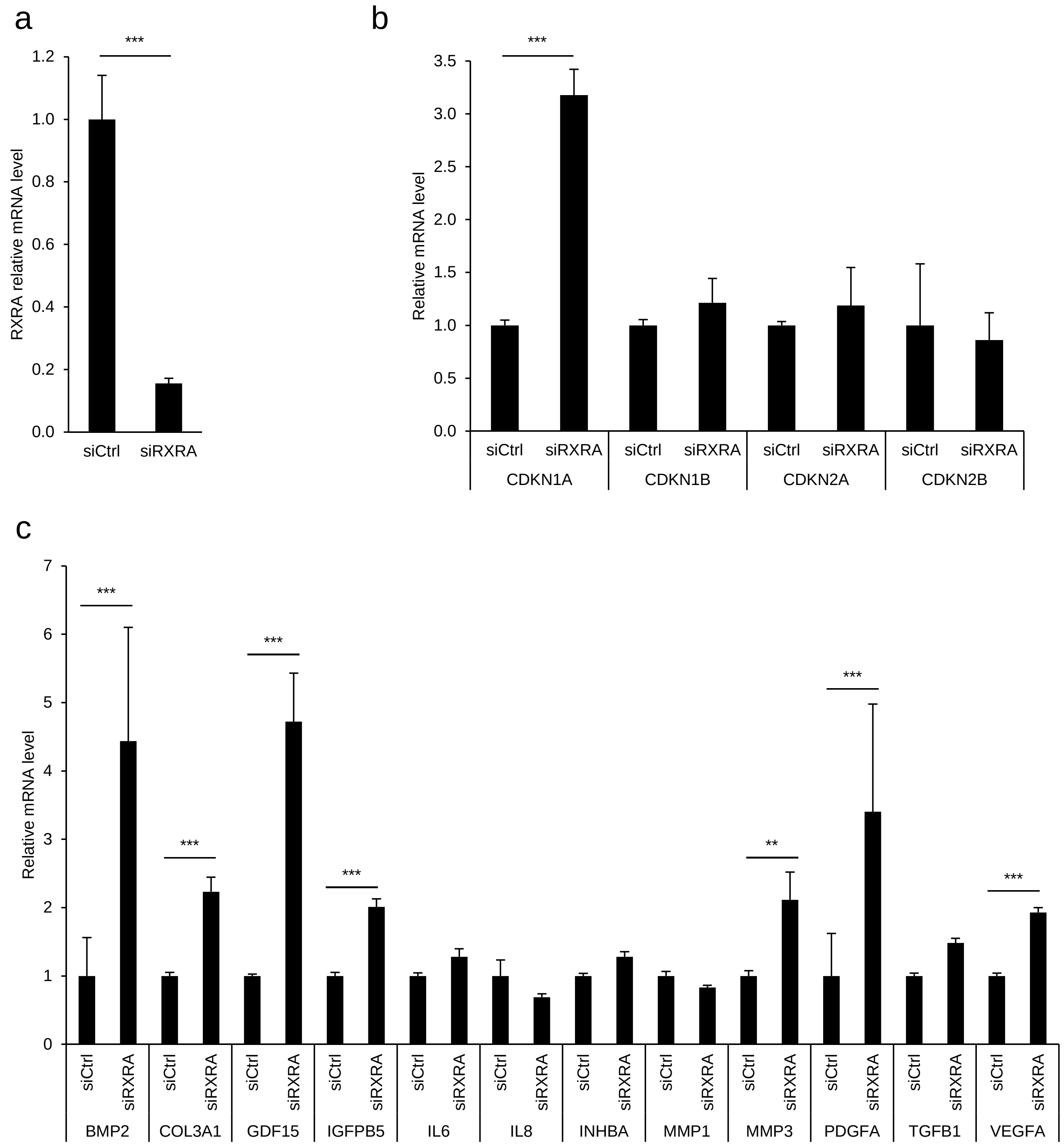

Supplementary Figure 6 RXRA knockdown induces the expression of CDKN1A and of components of the senescence-associated secretory phenotype (SASP). MRC5 cells were transfected with a control non-targeting siRNA pool (siCtrl) or a siRNA pool targeting RXRA (siRXRA). 4 days later, RXRA knockdown efficiency was confirmed (a) and mRNA levels of cyclin-dependent kinases inhibitors (b) and SASP components (c) were analyzed by RT-qPCR. The experiments shown are representative of at least three biological replicates. Statistical analysis was performed with Student’s t-test (** for P <0.01, *** for P <0.001).

Supplementary Figure 7, Ma et al

a

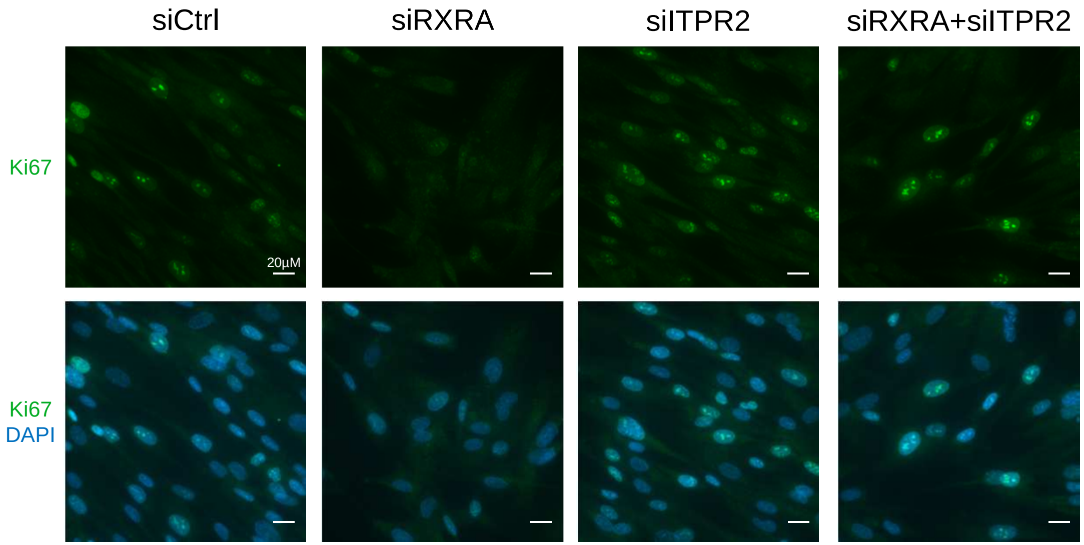

b

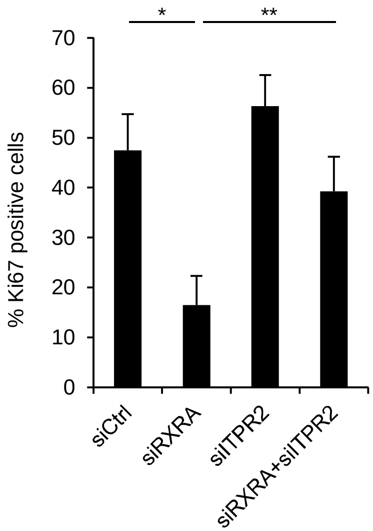

Supplementary Figure 7 RXRA knockdown triggers an ITPR2-dependent decrease in Ki67 proliferation index. MRC5 cells were transfected with a control non-targeting pool (siCtrl) or siRNA pools targeting RXRA or ITPR2 as indicated. 6 days after transfection, Ki67 staining by immunofluorescence was performed. Representative pictures (a) and the percentage of Ki67-positive cells counted in each condition (b) are shown. The experiments shown are representative of at least two biological replicates. Statistical analysis was performed with Student's t-test (* for P <0.05, ** for P <0.01).

Supplementary Figure 8, Ma et al

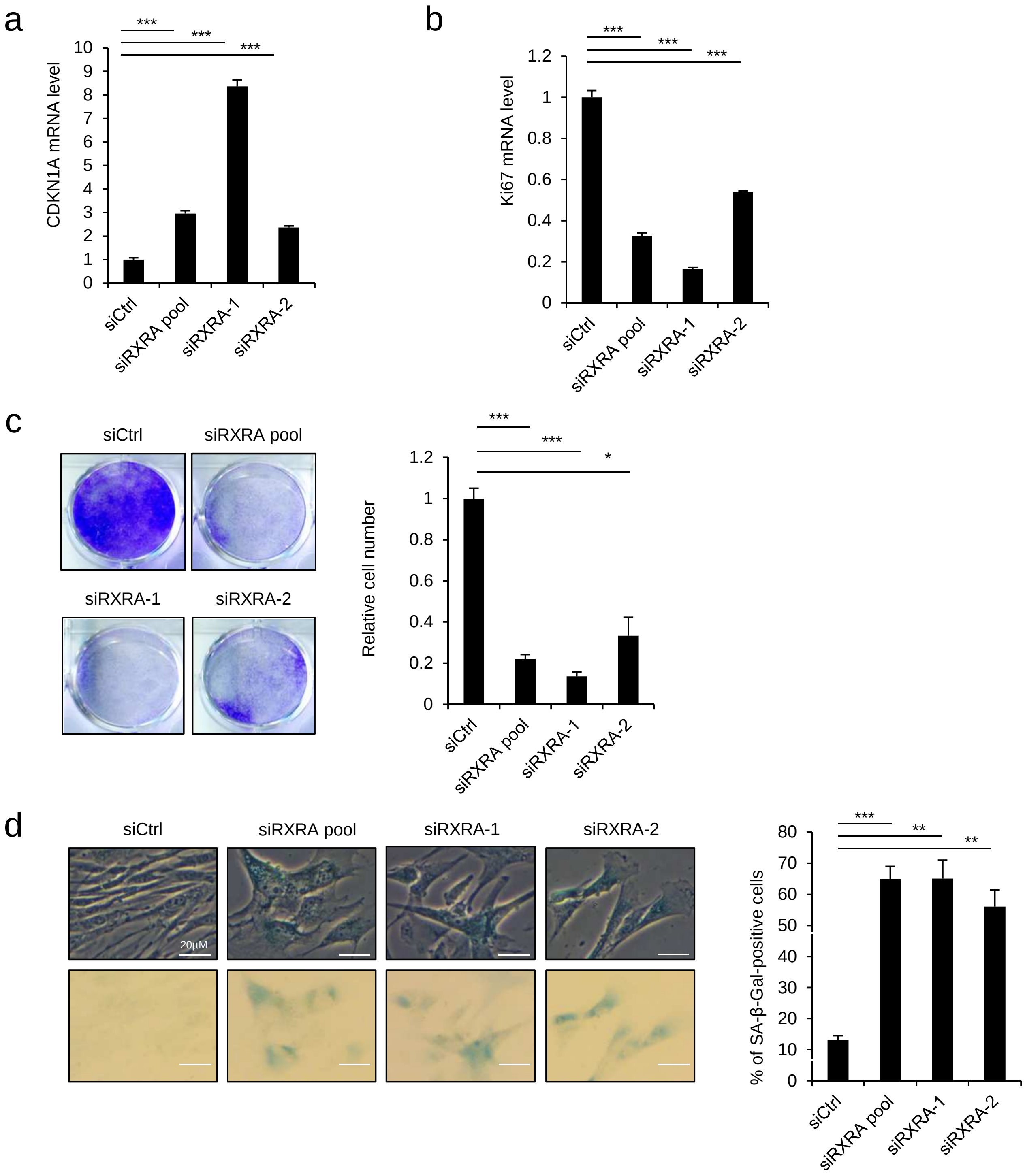

Supplementary Figure 8 RXRA knockdown induces cellular senescence in MRC5. MRC5 primary human lung fibroblasts were transfected with a control non-targeting siRNA pool (siCtrl), a siRNA pool targeting RXRA (siRXRA pool) or individual siRNAs targeting RXRA (RXRA-1 and RXRA-2). Knockdown efficiency was confirmed as shown in Supplementary Figure 2a. (a-b) 4 days after transfection CDKN1A (a) and Ki67 (b) mRNA levels were checked by RT-qPCR. (c) 6 days after transfection, cells were stained with crystal violet (left) and counted (right). (d) SA-β-galactosidase assay was also performed 6 days after transfection. Representative pictures are shown (left) as well as the percentage of SA-β-galactosidase positive cells counted in each condition (right). The experiments shown are representative of at least two biological replicates. Statistical analysis was performed with Student's t-test (* for $P < 0.05$, ** for $P < 0.01$, *** for $P < 0.001$).

Supplementary Figure 9, Ma et al

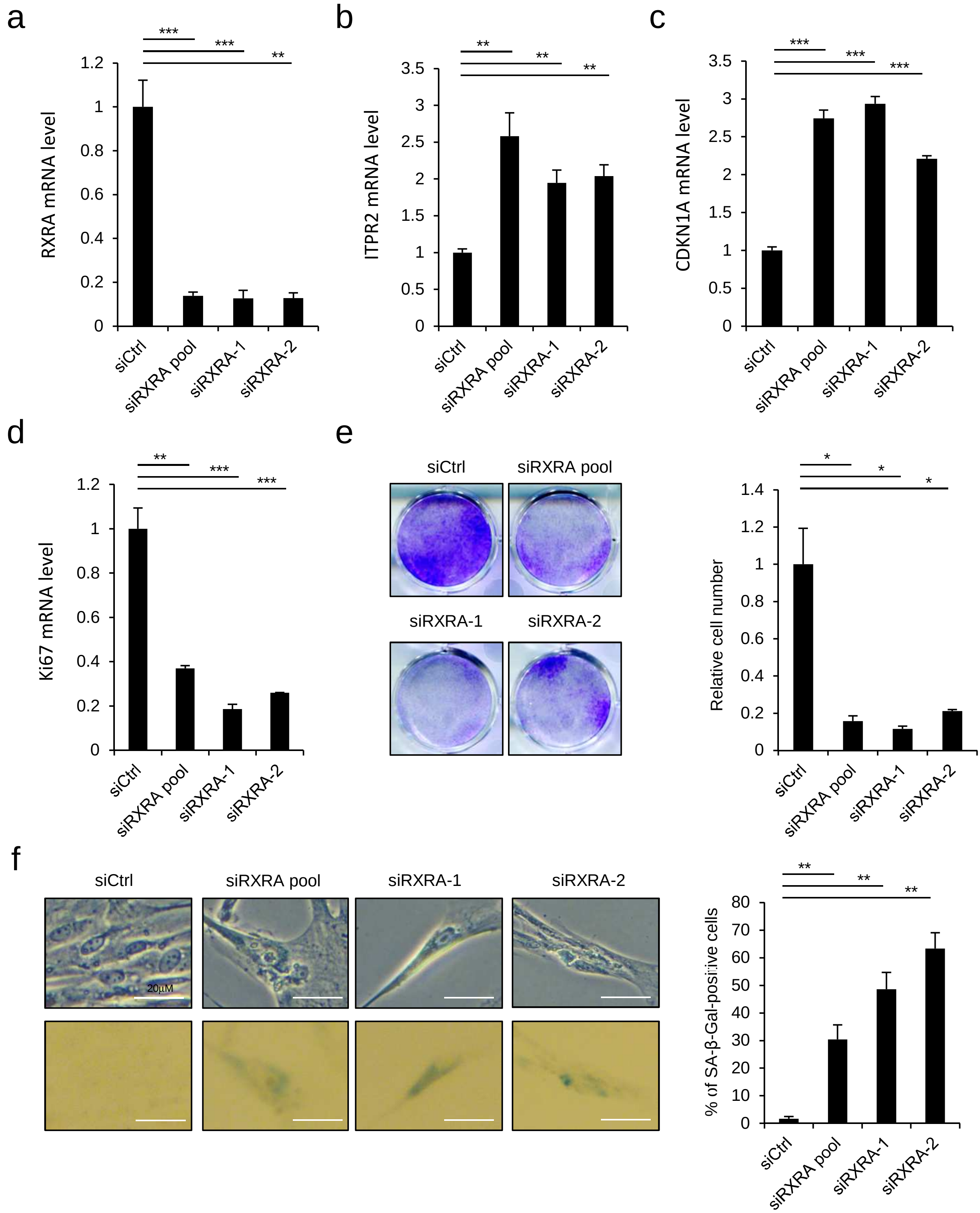

Supplementary Figure 9 RXRA knockdown induces cellular senescence in IMR90. IMR90 primary human lung fibroblasts were transfected with a control non-targeting siRNA pool (siCtrl), a siRNA pool targeting RXRA (siRXRA pool) or individual siRNAs targeting RXRA (RXRA-1 and RXRA-2). (a-d) RXRA (a), ITPR2 (b), CDKN1A (c) and Ki67 (d) mRNA levels were checked by RT-qPCR 4 days after transfection. (e) Cells were stained with crystal violet (left) and counted (right) 6 days after transfection. (f) SA-β-galactosidase assay was also performed 6 days after transfection. Representative pictures (left) and the percentage of SA-β-galactosidase positive cells counted in each condition (right) are shown. The experiments shown are representative of at least two biological replicates. Statistical analysis was performed with Student's t-test (* for P <0.05, ** for P <0.01, *** for P <0.001).

Supplementary Figure 10, Ma et al

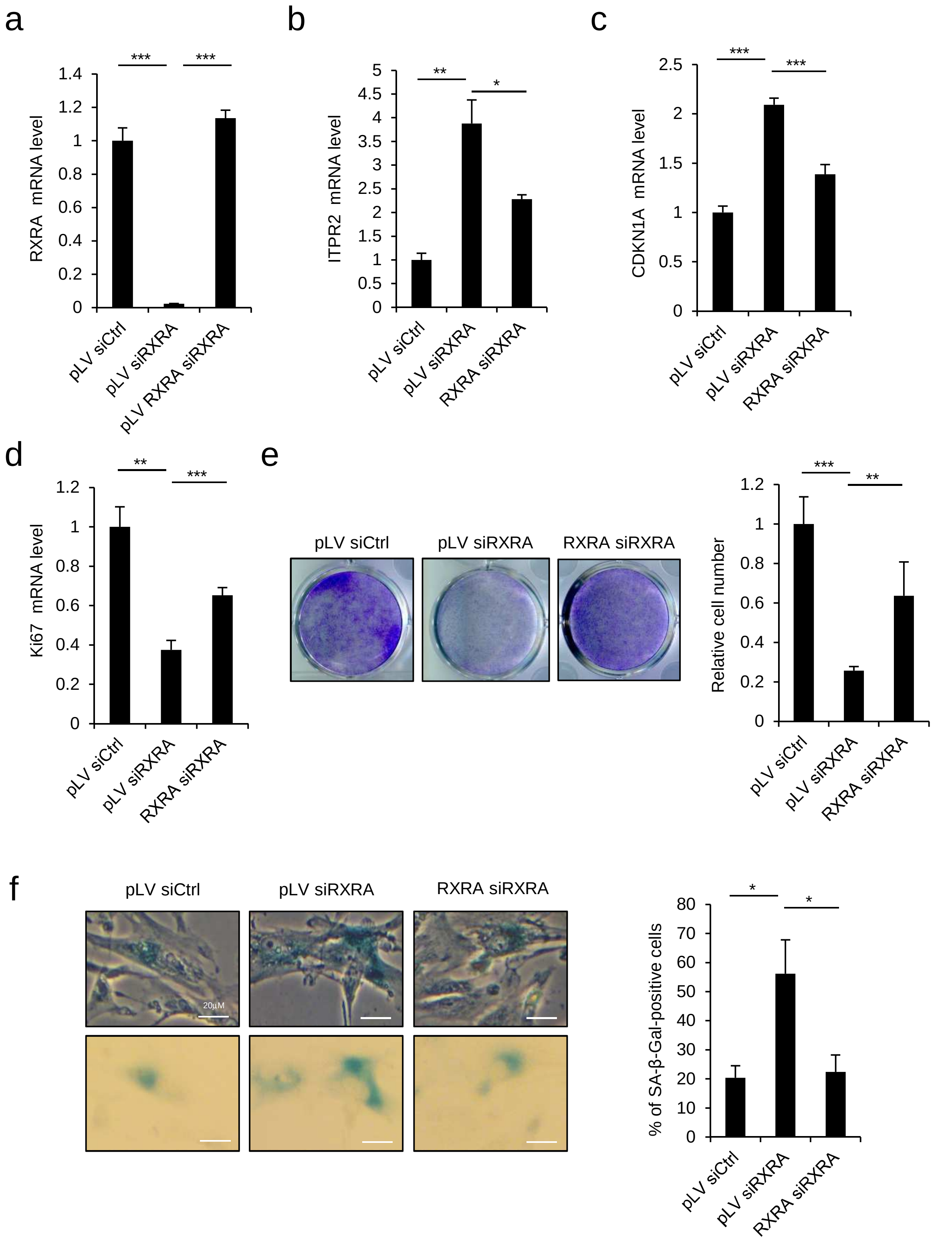

Supplementary Figure 10 Cellular senescence induced by RXRA knockdown is rescued by overexpressing RXRA. MRC5 cells were infected with a lentiviral vector encoding RXRA (RXRA) or the corresponding empty vector as control (pLV), and then transfected with a control non-targeting siRNA pool (siCtrl) or a siRNA pool targeting RXRA (siRXRA pool) as indicated. (a-d) 4 days after transfection, RXRA (a), ITPR2 (b), CDKN1A (c) and Ki67 (d) mRNA levels were checked by RT-qPCR. (e) 6 days after transfection, cells were stained with crystal violet (left) and counted (right). (f) SA-β-galactosidase assay was also performed 6 days after transfection. Representative pictures (left) are shown as well as the percentage of SA-β-galactosidase positive cells counted in each condition (right). The experiments shown are representative of at least two biological replicates. Statistical analysis was performed with Student's t-test (* for P <0.05, ** for P <0.01, *** for P <0.001).

Supplementary Figure 11, Ma et al

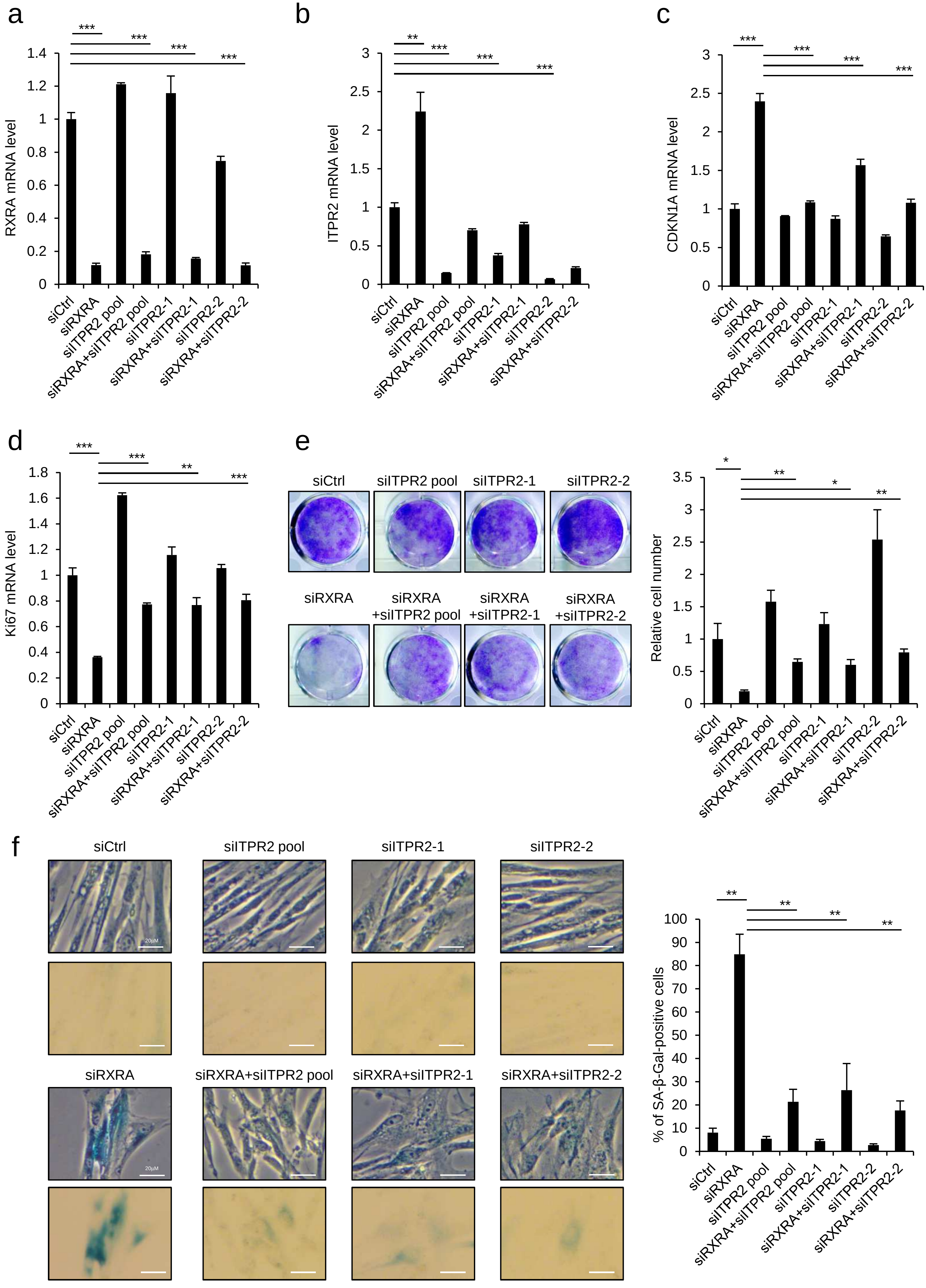

Supplementary Figure 11 RXRA knockdown induces cellular senescence in an ITPR2 dependent-manner. MRC5 cells were transfected with a control non-targeting pool (siCtrl), a siRNA pool targeting RXRA (siRXRA) and a siRNA pool (siITPR2 pool) or individual siRNAs (siITPR2-1 and siITPR2-2) targeting ITPR2, as indicated. (a-d) 4 days after transfection, mRNA levels of RXRA (a), ITPR2 (b), CDKN1A (c) and Ki67 (d) were checked by RT-qPCR. (e) 6 days after transfection, cells were stained with crystal violet (left) and counted (right). (f) SA-β-galactosidase assay was also performed 6 days after transfection. Representative pictures are shown (left) as well as the percentage of SA-β-galactosidase positive cells counted in each condition (right). The experiments shown are representative of at least two biological replicates. Statistical analysis was performed with Student’s t-test (* for $P < 0.05$, ** for $P < 0.01$, *** for $P < 0.001$).

Supplementary Figure 12, Ma et al

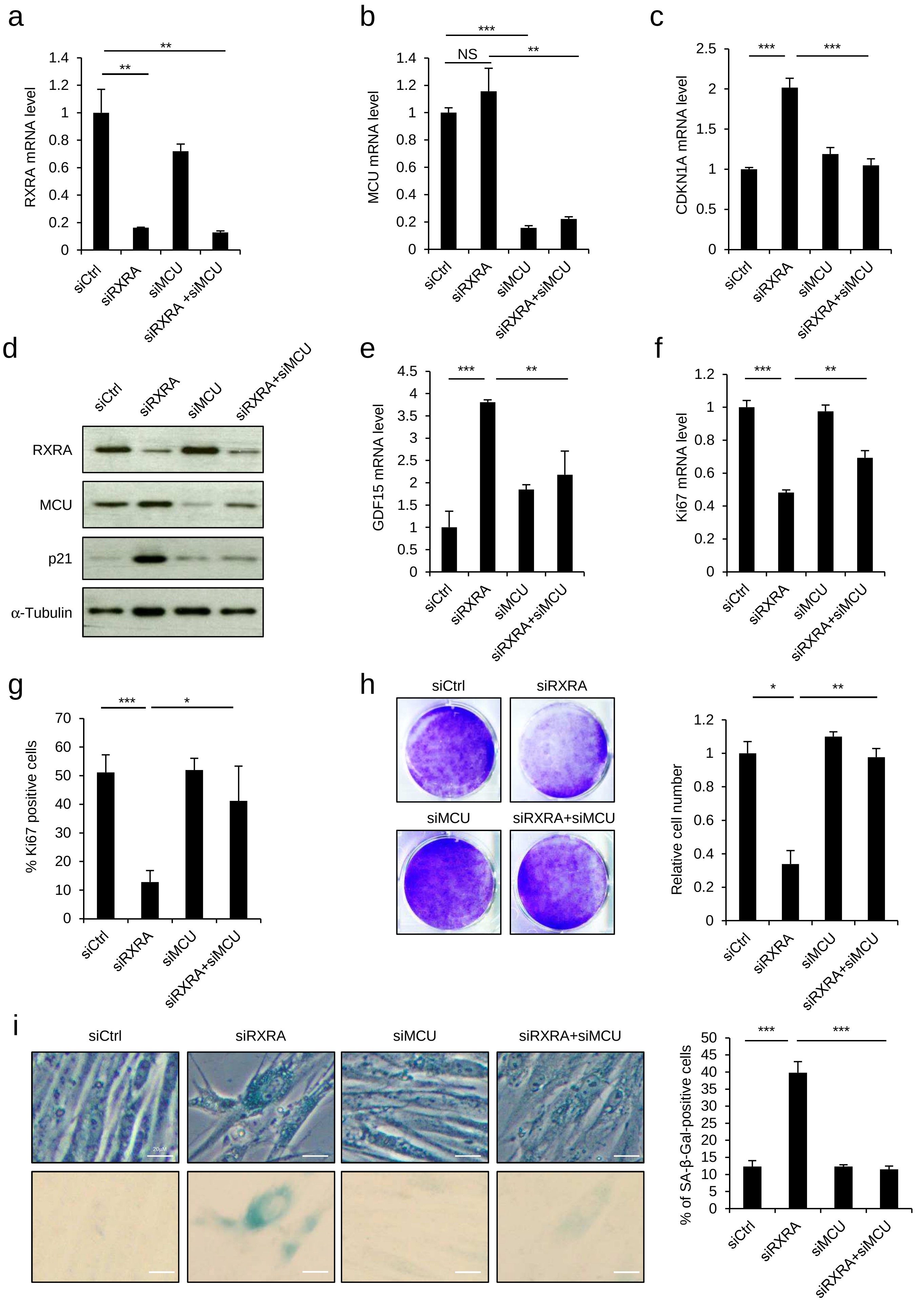

Supplementary Figure 12 Cellular senescence induced by RXRA knockdown depends on MCU. MRC5 cells were transfected with a control non-targeting pool (siCtrl) or siRNA pools targeting RXRA or MCU as indicated. (a-c) 4 days later, mRNA levels of RXRA (a), MCU (b) and CDKN1A (c) were checked by RT-qPCR. (d) RXRA, MCU and p21 protein levels were analyzed by Western Blot 6 days after transfection. β-tubulin was used as loading control. (e-f). GDF15 (e) and Ki67 (f) mRNA levels were also checked by RT-qPCR 4 days after transfection. (g) Ki67 staining by immunofluorescence was performed 6 days after transfection. (h) Cells were stained with crystal violet (left) and counted (right) 6 days after transfection. (i) SA-β-galactosidase assay was also performed 6 days after transfection. Representative pictures (left) and the percentage of SA-β-Gal positive cells counted in each condition (right) are shown. The experiments shown are representative of at least three biological replicates. Statistical analysis was performed with Student's t-test (* for $P < 0.05$, ** for $P < 0.01$, *** for $P < 0.001$).

Supplementary Figure 13, Ma et al

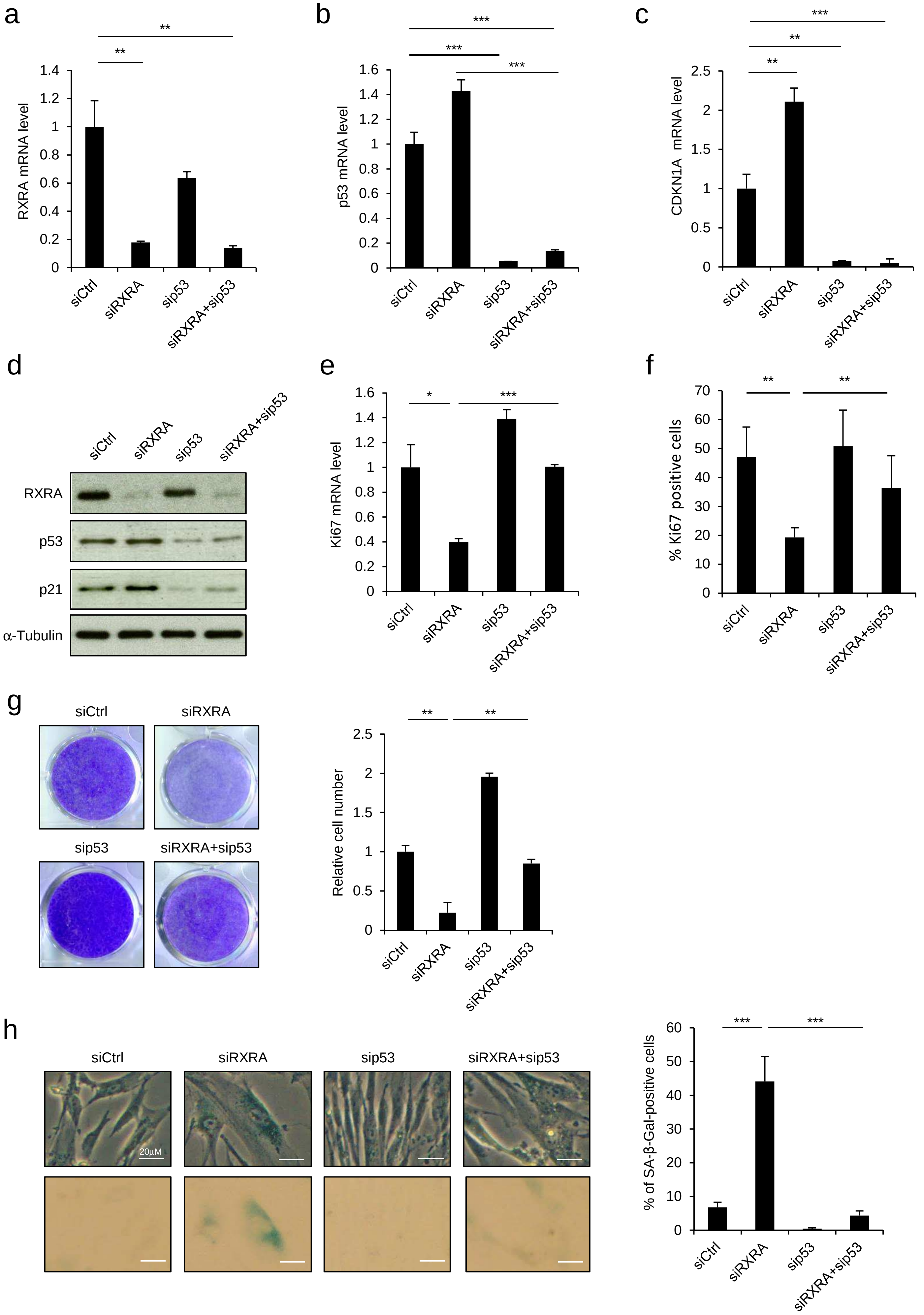

Supplementary Figure 13 RXRA knockdown triggers p53-dependent cellular senescence. MRC5 cells were transfected with a control non-targeting pool (siCtrl) or siRNAs targeting RXRA or p53 as indicated. (a-c) 4 days later, RXRA (a), p53 (b) and CDKN1A (c) mRNA levels were checked by RT-qPCR. (d) Protein levels of RXRA, p53 and p21 were analyzed by Western Blot 6 days after transfection. α-tubulin was used as loading control. (e-f). Ki67 was analyzed at the mRNA level by RT-qPCR 4 days after transfection (e) and at the protein level by immunofluorescence staining 2 days later (f). (g) 6 days after transfection, cells were stained with crystal violet (left) and counted (right). (h) SA-β-galactosidase assay was also performed 6 days after transfection. Representative pictures (left) and the percentage of SA-β-Gal positive cells counted in each condition (right) are shown. All the experiments shown are representative of at least three biological replicates. Statistical analysis was performed with Student's t-test (** for $P < 0.01$, *** for $P < 0.001$).

Supplementary Figure 14, Ma et al

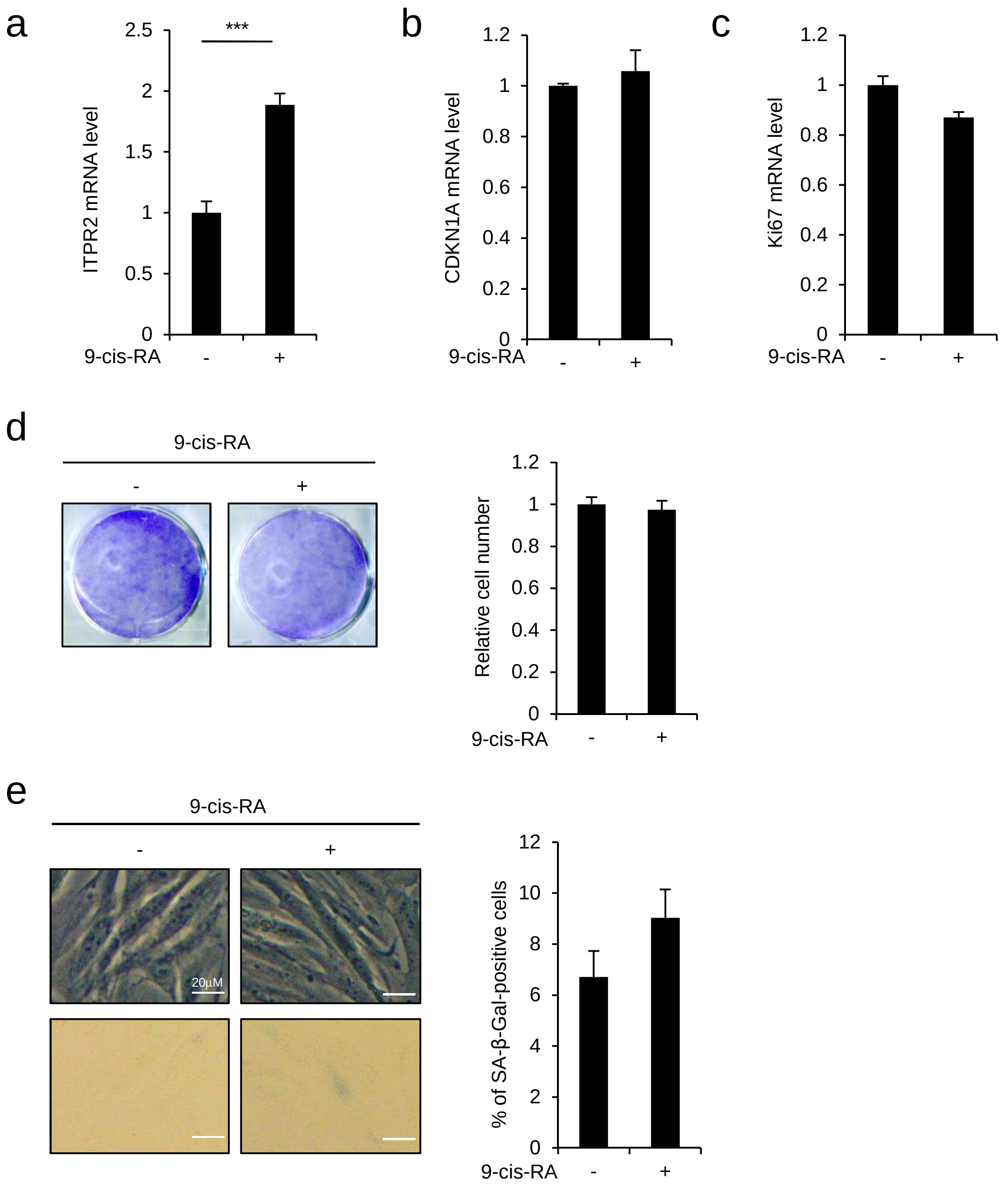

Supplementary Figure 14 9-cis-retinoic acid, a RXRA agonist, induces ITPR2 expression but does not induce cellular senescence in MRC5. MRC5 cells were treated with 500 nM 9-cis-retinoic acid (9-cis-RA) every two days where indicated. (a) At day 4, mRNA levels of ITPR2 (a), CDKN1A (b) and Ki67 (c) were analyzed by RT-qPCR. (d) At day 6, crystal violet staining (left) and cell counting (right) were performed. (e) SA-β-galactosidase assay was also performed after 6 days. Representative pictures (left) and the percentage of SA-β-Gal positive cells counted in each condition (right) are shown. All the experiments shown are representative of at least three biological replicates. Statistical analysis was performed with Student's t-test (*** for P <0.001).
