# Supplementary material for: The nuclear receptor RXRA controls cellular senescence by regulating calcium signaling
Source: Aging Cell. 2018 Sep 14;17(6):e12831. doi: 10.1111/acel.12831 (PMC6260923; doi:10.1111/acel.12831)
Supplement: Supplementary file 2 [file ACEL-17-e12831-s002.pdf]

Supplementary Table 1, Ma et al

siRNA	ITPR2 mRNA level	siRNA	ITPR2 mRNA level	siRNA	ITPR2 mRNA level	siRNA	ITPR2 mRNA level
BRD4	0,390	PCGF5	0,869	CBX1	1,104	TET2	1,541
KDM4A	0,506	KMT2A	0,873	PCGF6	1,105	SETD1B	1,580
SMARCD1	0,514	MECP2	0,874	BRD3	1,111	YTHDF3	1,615
RBBP4	0,553	SETD8	0,884	KDM5B	1,115	ZNF217	1,621
CHD6	0,560	REST	0,886	SMARCC1	1,122	ZHX2	1,624
RCOR1	0,617	ELP3	0,886	KMT2E	1,126	SMAD4	1,627
CHD4	0,630	CBX7	0,888	KDM6B	1,127	SUV420H2	1,662
KAT6B	0,639	HDAC4	0,892	CHD8	1,133	ZNF695	1,666
HAT1	0,660	KDM5A	0,897	PHC2	1,171	SMCHD1	1,708
MBD6	0,668	KMT2C	0,920	HDAC7	1,174	ZBTB33	1,718
SMARCA2	0,675	LMNB2	0,926	EHMT2	1,176	MPHOSPH8	1,722
KDM1A	0,681	SETD1A	0,928	KDM5C	1,183	DLX5	1,729
KDM2B	0,684	KDM4D	0,931	KDM1B	1,185	ZHX1	1,730
KDM2A	0,688	PRDM2	0,932	SUV420H1	1,202	CTBP2	1,731
HDAC3	0,709	BRD7	0,935	DNMT3A	1,204	TRIM28	1,746
PHC1	0,721	RING1	0,952	SIRT6	1,219	ARID1A	1,771
KAT2B	0,727	HDAC11	0,953	SIN3A	1,225	TDG	1,776
SMARCA5	0,729	HDAC1	0,973	ASH1L	1,228	ASF1A	1,781
SIRT2	0,735	SIRT3	0,975	CBX6	1,231	TRIM24	1,788
MBD3	0,737	PHF8	0,976	KDM3B	1,238	SUV39H1	1,804
KAT6A	0,738	PCGF3	0,977	CBX3	1,247	ZBTB40	1,815
DICER1	0,739	CHD3	0,980	MBD1	1,251	ZBTB4	1,834
SIRT1	0,739	BMI1	0,994	HMGB3	1,251	SMARCB1	1,859
PPM1D	0,740	EZH2	0,997	PBRM1	1,251	ZNF416	1,881
MBD5	0,743	SIRT7	1,025	SMARCC2	1,252	YTHDF1	1,884
MBD2	0,778	SCMH1	1,028	SMARCA1	1,263	WTAP	1,888
LMNA	0,794	CHD1	1,030	YTHDF2	1,278	SUZ12	1,931
SETD7	0,795	KDM3A	1,031	CBX5	1,283	KLF5	1,945
HDAC8	0,804	EZH1	1,033	HMGB1	1,305	ZNF114	1,983
RBBP7	0,804	KDM4C	1,035	SETD2	1,310	UHRF2	1,993
HDAC6	0,807	KDM4E	1,045	ZCCHC7	1,310	ZBTB38	2,012
PCGF1	0,843	MBD4	1,046	CDYL2	1,381	RXRA	2,076
SETDB2	0,843	HDAC2	1,048	KAT2A	1,400	TET3	2,310
CDYL	0,846	BRD1	1,057	TRIM33	1,417	ZBTB44	2,315
BRD2	0,847	PRMT5	1,060	DNMT3B	1,445	YTHDC1	2,383
EED	0,850	AZI2	1,072	SMARCA4	1,465	CTBP1	2,402
INO80	0,855	PCGF2	1,075	BRD8	1,484	SCAPER	2,440
SETDB1	0,860	BPTF	1,085	SMARCD2	1,495	UHRF1	2,506
PRMT1	0,866	ATRX	1,086	HOXA5	1,505	MEIS1	2,516
RNF2	0,866	DNMT1	1,087	HDAC5	1,510	ZBTB17	2,582

Supplementary Table 1. siRNAs used in the screen and corresponding ITPR2 mRNA level measured by Nanostring, relative to ITPR2 mRNA level measured for siControl set at 1. siRNAs inducing an upregulation of ITPR2 mRNA level of more than 2 fold compared to siControl are highlighted in red.

Supplementary Table 2, Ma et al

siRNA pool name	siRNA sequences
Control	UGGUUUACAUGUCGACUAA
	UGGUUUACAUGUUGUGUGA
	UGGUUUACAUGUUUUCUGA
	UGGUUUACAUGUUUUCCUA
RXRA	GCGCCAUCGUCCUCUUUAA
	GCAAGGACCGGAACGAGAA
	AGACCUACGUGGAGGCAAA
	UCAAAUGCCUGGAACAUCU
ITPR2	CGAAAUGGCCGCUCUAUUA
	GCAAUGGGUUGGAGACUAU
	GGAAUGAAAGGGCAAUUAA
	GAGCAAUAACUACCGGAUU
MCU	GAUCAGGCAUUGUGGAAUA
	GUUUUGACCUAGAGAAAUA
	ACUGAGAGACCCAUUACAA
	GUAAUGACACGCCAGGAAU
p53	GAAAUUUGCGUGUGGAGUA
	GUGCAGCUGUGGGUUGAUU
	GCAGUCAGAUCCUAGCGUC
	GGAGAAUAUUUCACCCUUC

Supplementary Table 2. Sequences of siRNAs in siRNA pools used in this study (4 siRNAs per pool).

Supplementary Table 3, Ma et al

siRNA name	siRNA sequences
RXRA-1	GCGCCAUCGUCCUCUUUAA
RXRA-2	AGACCUACGUGGAGGCAAA
ITPR2-1	CGAAAUGGCCGCUCUAUUA
ITPR2-2	GCAAUGGGUUGGAGACUAU

Supplementary Table 3. Sequences of individual siRNAs used in this study.

Supplementary Table 4, Ma et al

Symbol	Note	Accession #	Probe Name	Target Sequence
ITPR2	ORF of interest	NM_002223.2	NM_002223.2:7390	GTTTTGTTGGAAATCGTGGCACGTTCACCCGTGGGTACCGAGCAGTCATCC TGGATATGGCCTTTCTCTATCACGTGGCGTATGTCCTGGTTTGCATGCT
PGK1	Normalizer	NM_000291.3	NM_000291.3:964	ATTGTCAAAGACCTAATGTCCAAAGCTGAGAAGAATGGTGTGAAGATTACC TTGCCTGTTGACTTTGTCACTGCTGACAAGTTTGATGAGAATGCCAAGA
TBP	Normalizer	NM_001172085.1	NM_001172085.1:587	ACAGTGAATCTTGGTTGTAAACTTGACCTAAAGACCATTGCACTTCGTGCCC GAAACGCCGAATATAATCCCAAGCGGTTTGCTGCGGTAATCATGAGGA
TUBB2A	Normalizer	NM_001069.2	NM_001069.2:1410	AGGACGAGGCTTAAAAACTTCTCAGATCAATCGTGCATCCTTAGTGAACTTC TGTTGTCCTCAAGCATGGTCTTTCTACTTGTAAACTATGGTGCTCAGT
NEG_A	internal negative control	ERCC_00096.1	ERCC_00096.1:230	AACCGCCGCATACGGCCGATTGTCGCAGCCCGGGTCGATTATAACAACGG TGCAATCTCAGCTAAACCGACGCAGTTTTGCTCCTTGGATTCTGAGCCCG
NEG_B	internal negative control	ERCC_00041.1	ERCC_00041.1:440	GCACTGGCATTGGTCGTTTCAGGAGGCCATACAGAACTGGTTTATATGAAG GAACATGGATCATTTGAAGTCATTGGGGAAACCCTTGATGATGCGGCAG
NEG_C	internal negative control	ERCC_00019.1	ERCC_00019.1:140	GTACAGGCTGCTGGCTCATGTTTCCTTCTACGCTGCACTTGCGGGCATAGA GGTCGGTTGCGATCTATATTCGGAGATAACTATTCACCCAGCGCCACTC
NEG_D	internal negative control	ERCC_00076.1	ERCC_00076.1:355	AGAGATCACGTGGACCAAAGCTGATTGATTACGGGACTGGCCGTAAGTGC TGCCCGCGAGTAGATCGTCTAGATCCGGCTAAAATTCCCTGCGGTGCCTT
NEG_E	internal negative control	ERCC_00098.1	ERCC_00098.1:785	CCCAGATGACCTTCTCCCTCATAATCACTTAATCTGAGCGCAGGAGGCAGG CTGTATTAATTCCGGCCTCCAACCGGACCGTGGAACGACGCGACCAAGT
NEG_F	internal negative control	ERCC_00126.1	ERCC_00126.1:220	GGGTCCTTACCGGCTGTAAGCTCACTACAATCCAGGTACAGAGTGCGTTAA CCGGCCATTAGAGGGCCGCTACACCCGTCAGAATTTAAACGTATGGGCG
NEG_G	internal negative control	ERCC_00144.1	ERCC_00144.1:15	ACCCGTATGAACTGTTGCCGGCTCGGAAATGTTAAGGCTCTGCGCACGCA CTTTATCATTCGCAGCCTGTTCTGTCAGCGGGTCAGCCTAGGTTACGGTG
NEG_H	internal negative control	ERCC_00154.1	ERCC_00154.1:115	TTGGTCCGAGGAGGCATATAGGAAACGATGGGCACGCGCTATTCAGACGT TATTTGGTATGGAGTAAGAGGCCGGAAACTGGGCTCGATTGATGGATACT
POS_A	internal positive control	ERCC_00117.1	ERCC_00117.1:385	TCAGGCCTTGCCCTTACTAATGGCGCGTTGTAACGGGCCTTGAGGGAATGT CACTATTGAGGCACCCGTTCGACCCTCAGAGATATACCATTCCGCCTAT
POS_B	internal positive control	ERCC_00112.1	ERCC_00112.1:695	ATGAAAGCGCTGCTACTATGATAAGAGTACACGTACAGGTCTCGCCCGATT GGATTATGGCGAGCTGCCGCATTGACGGACATACCTTTGAACGTAATCG
POS_C	internal positive control	ERCC_00002.1	ERCC_00002.1:850	GCCGCTTTCGCTCGGGTCTGCGGGTTATAGCTTTTCAGTCTCGACGGGCTA GCACACATCTGGTTGACTAGGCGCATAGTCGCCATTCACAGATTTGCTC
POS_D	internal positive control	ERCC_00092.1	ERCC_00092.1:540	TACCTGGCATTTGGGCACTTCTTGCGTTTAAGCGGGAAAGATCGCGAGGG CCCGCTATTTGCGATACTTCCCATGTCGGTGCCGTCGCCTCTATGTACTC
POS_E	internal positive control	ERCC_00035.1	ERCC_00035.1:485	GGTTGAATTTGAGCGGATGGGCTCAACTGCGTCGTAACCGGTAGATACAG GGCATACGAGCCTCCCTATTTAACGGCATCATCCCGCGTAGTGCTGGTCA
POS_F	internal positive control	ERCC_00034.1	ERCC_00034.1:195	ACGAAGCTGTTCGGCCGCACGCAAGTACCTCCCACTTAGAAAGCGAATAAC CCAACGACCGTGTTCAACCCTGGCCGTCTCTCAACCAGGTATGCAATCA

Supplementary Table 4. Sequences of the Nanostring probes.

Supplementary Table 5, Ma et al

Gene symbol	Forward primers	Reverse primers
PGK1	5’-cagctgctgggtctgtcat-3’	5’-gctggctcggctttaacc-3’
HPRT1	5’-tgaccttgatttattttgcatacc-3’	5’-cgagcaagacgttcagtcct-3’
RXRA	5’-acatgcagatggacaagacg-3’	5’-tcgagagccccttggagt-3’
ITPR2	5’-aaagcctcagtggaatcctgt-3’	5’-atggcaattccacgattttt-3’
MCU	5’-ctggctcccctggaaaag-3’	5’-ccaccccatagcaccaaa-3’
p53	5’-aggccttggaactcaaggat-3’	5’-cccttttttggacttcaggtg-3’
CDKN1A	5’-tcactgtcttgtacccttgtgc-3’	5’-ggcgtttggagtggtagaaa-3’
CDKN1B	5’-tttgacttgcatgaagagaagc-3’	5’-agctgtctctgaaaggacatt-3’
CDKN2A	5’-gtggacctggctgaggag -3’	5’-ctttcaatcggggatgtctg -3’
CDKN2B	5’-caacggagtcaaccgtttc-3’	5’-ggtgagagtggcagggtct-3’
Ki67	5’-tcaaggaactgattcaggagaag-3’	5’-gtgcactgaagaacacatttcc-3’
BMP2	5’- cggactgcggtctcctaa-3’	5’- ggaagcagcaacgctagaag-3’
COL3A1	5’-ctggaccccagggtcttc-3’	5’-gaccatctgatccagggtttc-3’
GDF15	5’-ccggatactcacgccaga-3’	5’-agagatacgcaggtgcaggt-3’
IGFBP5	5’-ctaccgcgagcaagtcaag-3’	5’-gtctcctcggccatctca-3’
IL6	5’-gatgagtacaaaagtcctgatcca-3’	5’-ctgcagccactggttctgt-3’
IL8	5’- gagcactccataaggcacaaa-3’	5’-atggttccttccggtggt-3’
INHBA	5’-agacagctcttaccacatgatacaa-3’	5’-tctcctcttcagcaaattctcttt-3’
MMP1	5’-tttgatgtaccctagctacacctc-3’	5’-ggattttgggaacgtccat-3’
MMP3	5’-gcagtttgctcagcctatcc-3’	5’-tttctcctaacaaactgtttcacatc-3’
PDGFA	5’-ggatacctcgcccatgttc-3’	5’-caggagtcgctggaggtc-3’
TGFB1	5’-actactacgccaaggaggtcac-3’	5’-tgcttgaacttgtcatagatttcg-3’
VEGFA	5’-ctacctccaccatgccaagt-3’	5’-ccatgaacttcaccacttcgt-3’
BMI1	5’-ccattgaattctttgaccagaa-3’	5’-ctgctgggcatcgtaagtatc-3’
CBX7	5’-cgtcatggcctacgagga-3’	5’- tgggtttcggacctctctt-3’
DNMT1	5’-caaaccccctttccaaacctc-3’	5’-taatcctggggctaggtgaa-3’
EED	5’-aatccggttgttgcaatctt-3’	5’-cagaggatggctcgtattgc-3’
EZH2	5’-ccgctgaggatgtggatac-3’	5’-cagtgtgcagcccacaac-3’
PHC1	5’-cacccctcaaccagtctcag -3’	5’- gggttcggtttacctgcaa-3’
PRDM2	5’- cctctgcaaatatgagagattctg-3’	5’-ctggaggcacctcctgact -3’
SUV39H1	5’-gaggtggatgccaggaaag-3’	5’-cccaattcttggcctgtact-3’
SUZ12	5’-acatgggagactattcttgatgg-3’	5’-gcaacgtaggtccctgagaa-3’
TET3	5’-gtgcctccttctcctttggt-3’	5’-ttccggagcacttcttcct-3’
UHRF1	5’-aagatccaggagctgttcca-3’	5’-aagagggtatggccgtcct-3’
MEIS1	5’-gcatgaatatgggcatgga-3’	5’-catactcccctggcatactttg-3’
ZBTB17	5’- agaagctgaggccgcttt-3’	5’- tcttgctccttttgctcctc-3’

Supplementary Table 5. Sequences of primers used for RT-qPCR.

Supplementary Table 6, Ma et al

Application	Antibody name	Reference	Company	Dilution
Western blot	RXRA	sc-553	Santa Cruz Biotechnology	1:500
	ITPR2	sc-398434	Santa Cruz Biotechnology	1:500
	MCU	HPA016480	Sigma-Aldrich	1:500
	p21	P1484	Sigma-Aldrich	1:500
	p53	sc-126	Santa Cruz Biotechnology	1:500
	α-Tubulin	T6199	Sigma-Aldrich	1:5000
Immunofluorescence	53BP1	4937	Cell Signaling Technology	1:300
	Ki67	sc-23900	Santa Cruz Biotechnology	1:300
Chromatin immunoprecipitation	RXRA	sc-553	Santa Cruz Biotechnology	1:125
	Normal rabbit IgG X	sc-2027 X	Santa Cruz Biotechnology	1:1250

Supplementary Table 6. Primary antibodies used in this study.

Supplementary Table 7, Ma et al

Name	Forward primers	Reverse primers
ITPR2.1	5’-AAACTTTCCTCCCTCGTTTACA-3’	5’-ATCCATGTTGGGTCCAGTTC-3’
ITPR2.2	5’-ACCAGGGCTTCTTTATGTTCTC-3’	5’-GTTAGGCAAACGATGGTAGGT-3’
ITPR2.3	5’-CCATTCATGTGGGCTGCTCT-3’	5’-CCTTTCCTTCCCAGGTGTTGA-3’
Positive control	5’-ATTCCCGGGCAATGACCAGA-3’	5’-GATCCCAAGGTTGCGTGGTC-3’
Negative control	5’- TTGAGCTCTGGCATAGAAGA -3’	5’-TACCCAGACACACTCTAAGG-3’

Supplementary Table 7. Sequences of qPCR primers used in chromatin immunoprecipitation experiments. Location of the amplified regions is indicated in Supplementary Figure 2c.
